# Cytosine epigenetic modification modulates the formation of an unprecedented G4 structure in the *WNT1* promoter

**DOI:** 10.1093/nar/gkz1207

**Published:** 2020-01-08

**Authors:** Zi-Fu Wang, Ming-Hao Li, I-Te Chu, Fernaldo R Winnerdy, Anh T Phan, Ta-Chau Chang

**Affiliations:** 1 Institute of Atomic and Molecular Sciences, Academia Sinica, Taipei 106, Taiwan, R.O.C; 2 Department of Chemistry, National Taiwan University, Taipei 106, Taiwan, R.O.C; 3 School of Physical and Mathematical Sciences, Nanyang Technological University, Singapore 637371, Singapore; 4 NTU Institute of Structural Biology, Nanyang Technological University, Singapore 636921, Singapore

## Abstract

Time-resolved imino proton nuclear magnetic resonance spectra of the WT22m sequence d(GGGCCACCGGGCAGTGGGCGGG), derived from the *WNT1* promoter region, revealed an intermediate G-quadruplex G4(I) structure during K^+^-induced conformational transition from an initial hairpin structure to the final G4(II) structure. Moreover, a single-base C-to-T mutation at either position C_4_ or C_7_ of WT22m could lock the intermediate G4(I) structure without further conformational change to the final G4(II) structure. Surprisingly, we found that the intermediate G4(I) structure is an atypical G4 structure, which differs from a typical hybrid G4 structure of the final G4(II) structure. Further studies of modified cytosine analogues associated with epigenetic regulation indicated that slight modification on a cytosine could modulate G4 structure. A simplified four-state transition model was introduced to describe such conformational transition and disclose the possible mechanism for G4 structural selection caused by cytosine modification.

## INTRODUCTION

A guanine-rich (G-rich) sequence is capable of forming a G-quadruplex (G4) via Hoogsteen hydrogen bonding under physiological condition (1–3). Compelling evidence suggests that G4s are involved not only in human telomeres for protecting the ends of chromosome but also in several gene promoters for regulating gene expression (4–6). Recently, several studies have highlighted that G4s are found in cells (7–11) and ligands inducing or stabilizing G4 structures indeed can inhibit cancer growth (12–17). However, the coexistence of various structures and the dynamic polymorphism of G4s of some G-rich sequences generally cause the challenges for structural analysis (18–22). Determination of G4 structure can provide invaluable information for better understanding of deep insight of DNA dynamics and rational designing of specific G4 ligands (20,23–26).

Previously (27), we have shown that a native G-rich sequence named WT22, 5′-GGGCCACCGGGCAGGGGGCGGG-3′, within the *WNT1* promoter region forms a hairpin (Hp) structure in the absence of K^+^ and converts into a G4 structure after addition of 150 mM K^+^. It is noted that WT22 can simultaneously adopt an Hp structure and various G4 structures in K^+^ solution. This is because the consecutive five G bases of WT22 allow the formation of intermolecular G4s, as also observed in the BCL2 promoter sequence bcl2mid (28). A single base mutation of WT22 at G/T(15), WT22m (Table 1), can eliminate the formation of intermolecular G4s and accelerate the transition from Hp to the same type of final unimolecular G4 structure of WT22 in 150 mM K^+^ solution (27).

**Table 1.** Oligonucleotide sequences used in this work and the corresponding Tm values

Name	Sequence(5′-3′)	*T*_m_
WT22m	GGGCC ACCGG GCAGT GGGCG GG	73.0°C
WT22m-T4	GGGTC ACCGG GCAGT GGGCG GG	69.2°C
WT22m-T5	GGGCT ACCGG GCAGT GGGCG GG	
WT22m-T7	GGGCC ATCGG GCAGT GGGCG GG	
WT22m-T8	GGGCC ACTGG GCAGT GGGCG GG	
WT22m-5mC4	GGG^5m^CC ACCGG GCAGT GGGCG GG	73.0°C
WT22m-5hmC4	GGG^5hm^CC ACCGG GCAGT GGGCG GG	69.5°C
WT22m-5fC4	GGG^5f^CC ACCGG GCAGT GGGCG GG	69.5°C
WT22m-5caC4	GGG^5ca^CC ACCGG GCAGT GGGCG GG	69.5°C

In this work, time-dependent imino proton nuclear magnetic resonance (NMR) spectra of WT22m reveal an intermediate G4(I) state existed during the conformational transition from an Hp structure to a final G4(II) structure after the addition of 150 mM K^+^ at 25°C. To determine the WT22m G4(I) structure, we found that the imino proton NMR spectra of a single-base mutation of WT22m at C/T(4) or C/T(7) show almost identical to the initial spectra of the WT22m G4(I), implying that a single-base mutation may lock G4(I) structure without further conformational change. Spectral analysis reveals that the G4(I) structure is different from the final G4(II) structure of WT22m. In addition, cytosine modification of WT22m by methylation and demethylation at C_4_ residue could cause significant difference in terms of structural populations between G4(I) and G4(II). A four-state transition model is applied to describe such conformational change, highlighting that the underlying G4 structural selection is linked to the difference of hydrogen bonding effect in loop configuration.

## MATERIALS AND METHODS

### DNA preparation

All unlabeled oligonucleotides were purchased from Bio Basic (Ontario, Canada), and oligonucleotides with cytosine modification were purchased from IBA (Goettingen, Germany). DNA concentrations were determined by absorption at 260 nm peaks using a UV-Vis absorption spectrometer (Nano-Viewer, GE Healthcare, USA). The oligonucleotides were dissolved in a buffer consisting of 10 mM Tris-HCl (pH 7.5) followed by heat-denaturation at 95°C for 5 min and slow annealing to 25°C (1 min/°C). The annealed oligonucleotides were stored at 4°C overnight prior to use. The site-specifically 6% ^15^N labeled oligonucleotides were synthesized using the solid-phase method as described previously (23).

### Circular dichroism spectroscopy

The CD experiments were conducted using a spectropolarimeter (J-815, Jasco, Japan) with a bandwidth of 2 nm, at the scanning speed of 50 nm/min and step resolution of 0.2 nm across the spectral range of 210–350 nm. The sample concentrations were 4 μM in 150 mM K^+^ solution overnight at 25°C. The thermal melting curves were recorded by a peltier thermal coupler chamber (PFD-425S/15, Jasco, Japan) and the molar *ellipticity* was monitored at 295 nm between 10 and 95°C with a temperature ramping rate of 1°C/min. The observed signals were baseline subtracted, and the first derivative lowest points were defined as the melting temperature.

### NMR spectroscopy

All NMR experiments were performed on a Bruker AVIII 800 MHz and AVIII 850 MHz spectrometers (Bruker, USA), which are equipped with a cryo-probe. The 1D imino proton NMR spectra were recorded by a WATERGATE pulsed sequence. The population of G4(I) and G4(II) are determined by the ratio of peak volume of imino-proton signal for several specific residues in which can be unambiguous assigned in both forms. The 1D ^15^N-^1^H SOFAST-HMQC spectra were used for unambiguous assignment of individual imino proton resonances using a series of site-specifically ^15^N-labeled NMR samples with 6% of ^15^N-labeled guanine. In the NMR experiments, the analyte concentrations were typically 0.1–0.2 mM for the 1D experiments and 0.5–1 mM for the 2D experiments in specific salt conditions with the internal reference of 0.1 mM 4,4-dimethyl-4-silapentane-1-sulfonic acid. Double quantum filtered Homonuclear Correlation (DQF-COSY), Total correlation (TOCSY) (mixing times of 50 and 150 ms) and ^1^H-^13^C HSQC spectra were used to cross-check the assignments of the NOEs. Through-bond correlations at natural abundance (H1/H8-C5) and heteronuclear multiple-bond correlation (JR-HMBC) were used to assign aromatic proton (H8). The NOESY spectra of exchange and non-exchange inter-proton were assigned using SPARKY software (UCSF). Inter-proton distances were calculated from the initial slopes of NOE buildup curves for NOESY spectra recorded at mixing times of 50, 100, 150, and 250 ms. The relative distance was calculated by using the cytosine H6-H5 fixed distance as the reference distance.

### Structure calculation

Structures were calculated based on distance geometry simulated annealing and distance-restrained molecular dynamics refinement using the XPLOR-NIH program. Hydrogen bond restraints, NOE distance restraints, dihedral restraints and planarity restraints were imposed during structure calculations. Structures were displayed using the Discovery studio 3.0 (Accerlys, USA) and the PyMOL program.

## RESULTS

### Time-resolved imino proton NMR spectra reveal two G4 structures of WT22m

**Figure 1.**
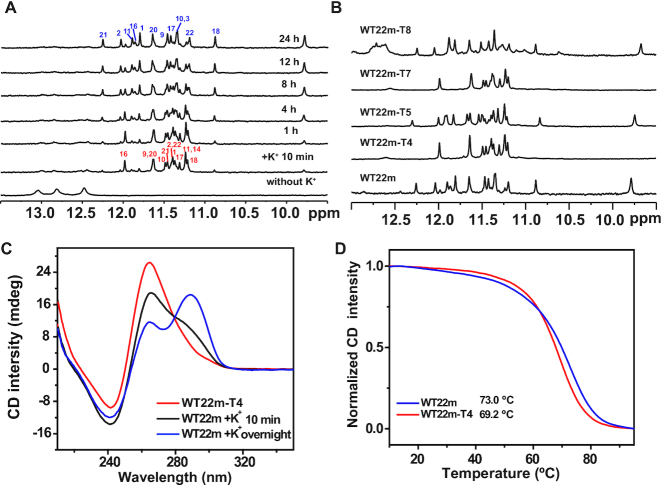
(**A**) Time-resolved imino proton spectra of WT22m recorded at 0, 10 min, 1, 4, 8, 12 and 24 h after the addition of 150 mM K^+^ at 25°C. (**B**) Imino proton NMR spectra of WT22m, WT22m-T4, WT22m-T5, WT22m-T7 and WT22m-T8 in 150 mM K^+^ solution overnight at 25°C. (**C**) CD spectra of WT22m in 150 mM K^+^ solution after 10 min (black line) and overnight (blue line) at 25°C, and WT22m-T4 (red line) in 150 mM K^+^ solution overnight at 25°C. (**D**) CD melting curves of WT22m (blue line) and WT22m-T4 (red line) in 150 mM K^+^ solution, monitored at CD 295- and 265-nm signal, respectively.

Time-dependent imino proton NMR spectra were applied to monitor the spectral change of WT22m after the addition of 150 mM K^+^. The results revealed the presence of an intermediate G4(I) state in the transition from an initial Hp state to a final G4(II) state after the addition of 150 mM K^+^ at 25°C (Figure 1A). In the initial transition, the imino proton resonances at 12.4–13.0 ppm rapidly reduce and several distinct imino proton resonances at 10.5–12.0 ppm appear concomitantly, implying the conformational change from an Hp structure to a G4 structure. Subsequently, a slow transition is followed to shift the equilibrium from the intermediate G4(I) to the final G4(II) topologies. Eventually, the imino proton NMR spectra overnight at 25°C are almost identical to the NMR spectrum obtained from an annealed WT22m.

To investigate the WT22m G4(I) structure, we performed single-base C-to-T mutation at positions 4, 5, 7 and 8 of WT22m (Table 1) to find if there is a spectral pattern similar to the imino proton NMR spectrum of WT22m G4(I). Among them, the NMR spectra of sequences with a single-base mutation at C/T(4) (WT22m-T4) or C/T(7) (WT22m-T7) showed almost identical imino proton signals to those of WT22m G4(I) (Figure 1B), suggesting that the substitution of C_4_ or C_7_ by a thymine could block the conformational conversion from the intermediate G4(I) state to the final G4(II) state of WT22m. In addition, circular dichroism (CD) of WT22m and WT22m-T4 in K^+^ solution showed different spectral patterns with WT22m exhibiting a major 295-nm band together with a minor 265-nm band and WT22m-T4 exhibiting a dominant 265-nm band (Figure 1C). For comparison, the CD profile of WT22m after addition of 150 mM K^+^ for 10 min showed a major 265-nm band associated with a shoulder around 295 nm (Figure 1C). The CD results of the spectral change from G4(I) to G4(II) of WT22m are consistent with the NMR results. The melting curves monitored by CD 295-nm signal for WT22m G4(II) and 265-nm signal for WT22m-T4 G4 showed that the melting temperature (*T*_m_) is ca. 73.0°C for WT22m G4(II), which is slightly higher than ca. 69.2°C for WT22m-T4 G4 (Figure 1D and Table 1).

### Unprecedented G4 structure of WT22m-T4

**Figure 2.**
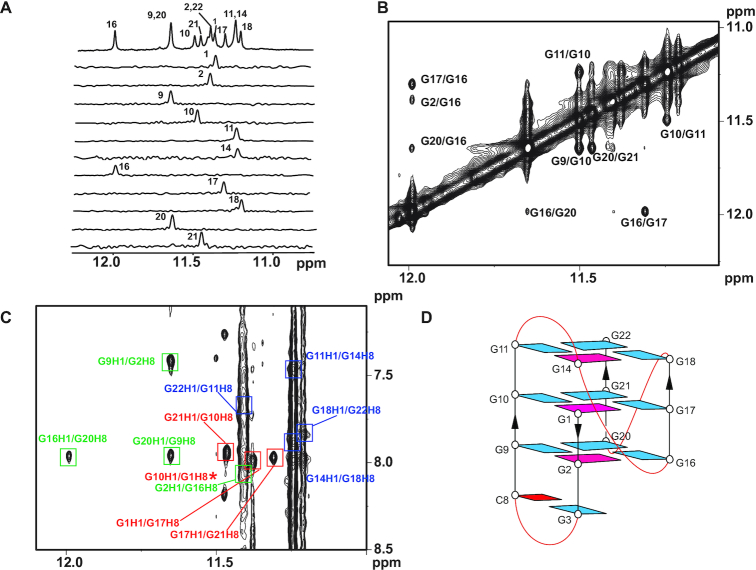
Determination of WT22m-T4 G4 structure in K^+^ solution. (**A**) Site-specific assignments of imino proton resonances of WT22m-T4 G4. The 1D ^15^N-^1^H SOFAST-HMQC spectra of 6% ^15^N enriched WT22m-T4 G4 samples are shown below with the assignments. (**B**) Guanine imino (H1)–imino (H1) proton and (**C**) imino (H1)–aromatic (H8) proton regions of NOESY spectrum of WT22m-T4 G4. The missing NOE (G10H1-G1H8) was marked with asterisks. The NOESY spectrum was recorded at 25°C with a mixing time of 250 ms. The cross peaks that correspond to the NOE connectives within the three G-quartets (colored green, red and blue) are boxed and labeled with the residue number of imino proton and that of aromatic proton. (**D**) Schematic representation of the WT22m-T4 G4(I) structure, where anti-guanines are colored in cyan, and syn-guanines are colored in magenta. Cytosine base with G-C pairing is colored red.

We proceeded to determine the G4 structure of WT22m-T4, which would mimic the structure of WT22m G4(I). Imino protons of each guanine of WT22m-T4 were unambiguously assigned by the site-specific ^15^N-labeled sample (Figure 2A). Surprisingly, the G_14_, within the C_12_-A_13_-G_14_-T_15_ segment, showed a distinct signal at ∼11.2 ppm characteristic of G4 formation, while the G_3_ from the first G-tract showed a distinct signal at ∼12.6 ppm characteristic of Watson–Crick base pair formation (Supplementary Figure S1). Moreover, guanine H8 proton assignments were obtained by ^1^H-^15^N HMQC in site-specific labeled samples (Supplementary Figure S2) and by ^1^H-^13^C JR-HMBC (29) of an unlabeled sample (Supplementary Figure S3). The spectral assignments for cytosine residues were completed by through-bond (DQF-COSY, TOCSY and ^1^H-^13^C HSQC) (data not shown) and through-space (NOESY) correlations between protons. According to the well-characterized NOEs between imino protons, imino and H8 protons, and H1′ and H8 protons (Figures 2B, 2C and S4), we established the G4 structure of WT22m-T4, involving three G-tetrads with all clockwise hydrogen-bonding directionality (from bottom view): G_14_→G_18_→G_22_→G_11_, G_1_→G_17_→G_21_→G_10_, and G_2_→G_16_→G_20_→G_9_ (Figure 2D). The glycosidic conformations of most guanines are anti, except those of G_1_, G_2_ and G_14_ are syn, as observed in the strong H1′-H8 NOE intensities by NOESY spectra with low mixing time (data not shown). In addition, sequences containing 8-Br-guanine substitutions at G_1_, G_2_ and G_14_ showed a similar imino proton spectral pattern to that of WT22m-T4 (Supplementary Figure S5A) and exhibited comparable melting temperature with that of WT22m-T4 (Supplementary Figure S5B) (30), consistent with these three residues adopting syn glycosidic conformations.

**Figure 3.**
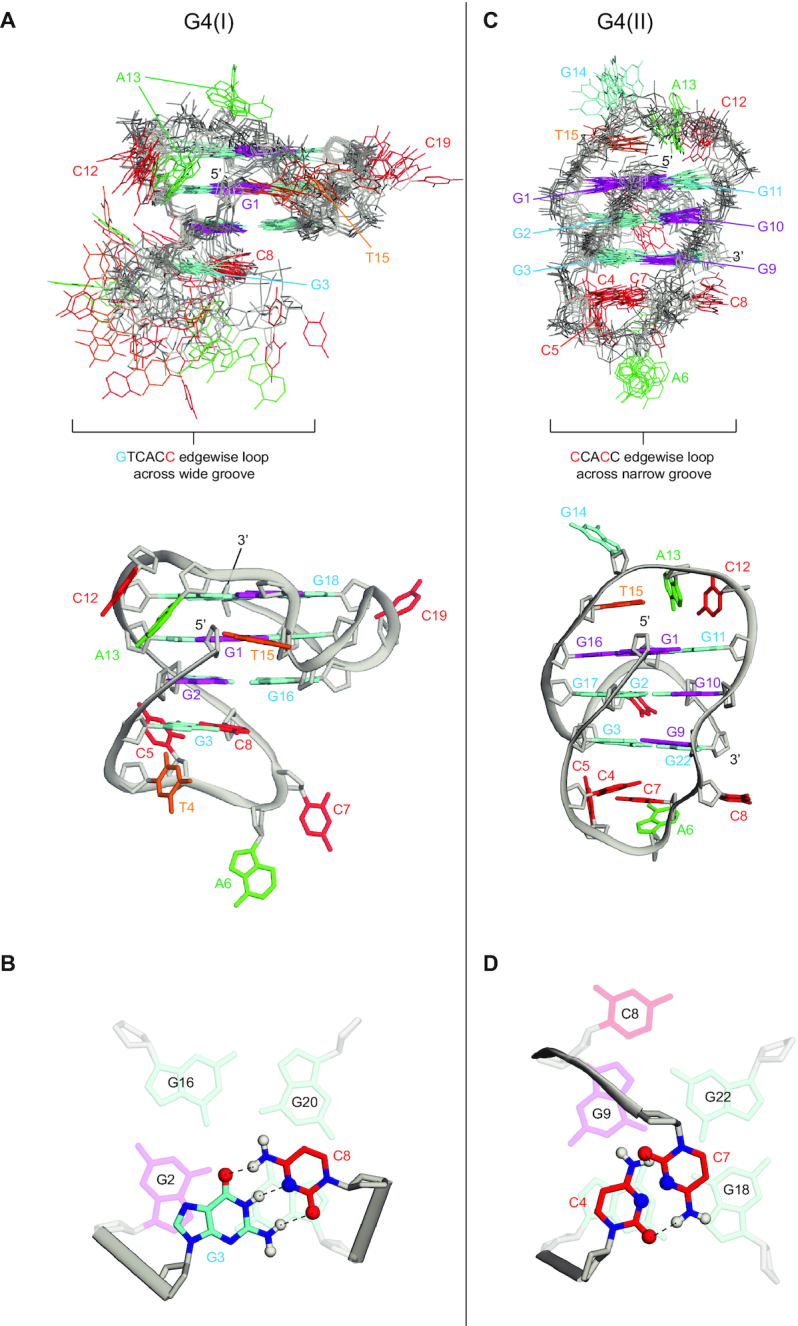
NMR structure of G4(I) and G4(II). (**A**) Ten superimposed refined structures of WT22m-T4 G4(I), and ribbon view of a representative structure. (**B**) G_3_•C_8_ Watson-Crick base pair of WT22m-T4 G4(I). (**C**) Ten superimposed refined structures of WT22m G4(II), and ribbon view of a representative structure. The anti and syn guanines are colored cyan and magenta, respectively; adenines are colored green; thymines, orange; backbone and sugar, gray. (**D**) C_4_•C_7_ base pair of WT22m G4(II). Hydrogen bonds between the base pairs were shown by black dotted lines.

The structure of WT22m-T4 G4 was computed on the basis of NMR restraints by the X-PLOR program (31). We began with 100 structures for structural refinement based on the previous protocol (32,33). Ten best structures which have lowest energy are superimposed in Figure 3A (Supplementary Table S1). The G4 structure of WT22m-T4 consists of four loops (Figure 3A), including a long lateral stem loop (G_3_ to C_8_) with G_3_ and C_8_ forming a Watson–Crick base pair (Figure 3B), a two-base edgewise loop C_12_-A_13_ to connect two adjacent corners for allowing the G_14_ taking part in the G-tetrad core, a single-base V-shaped loop (T_15_) and a propeller loop (C_19_) for bridging three G-tetrad layers. The hydrogen bonding of G_3_ and C_8_ explained why G_3_ was not involved in the G-tetrad core formation; replacing C_8_ by T_8_ can disrupt the current fold (data not shown). Such phenomenon of G-tract interacting with a loop base has been documented in a c-kit G4 structure (34). The continuous connection through the G4 wide groove between the G-tetrad core and a stem loop clamped by a G•C Watson–Crick base pair was previously shown to be one of the favorable quadruplex–duplex junctions (35).

### Interaction of cytosine residues stabilized G4(II) structure

Cytosine loops can be identified by observing the TOCSY, where the H6-H5 cross-peaks of cytosines are very strong. Here we showed the TOCSY spectrum of WT22m (Supplementary Figure S6A) to determine H6-H5 cross-peaks of all cytosine residue, C_4_, C_5_, C_7_, C_8_, C_12_ and C_19_, and also the correlation of H6-H4′ and H6-H4″ for the amino proton of C_4_. In addition, the H8/H6-H1′ NOE sequential connectivity of guanine residues and loop residues (Supplementary Figure S6B) was achieved with the assistance of TOCSY assignment. The H6-H5 cross peaks of C_4_, C_5_, C_7_, C_8_, C_12_ and C_19_ were confirmed by the strong intensity of intraresidue NOEs. Based on the assignment from TOCSY and H8/H6-H1′ NOE, we determined all cytosine H6-H5 NOEs of WT22m and found distinct amino proton signal of C_4_ around 9.7 and 8.2 ppm, indicating that an amino proton of C_4_ could form hydrogen bonding with nearby residue. Moreover, several NOEs of C_4_ and C_7_ were observed with the bottom G-tetrad (G_3_-G_9_-G_18_-G_22_) (Supplementary Figure S6C and D) suggested these two cytosine bases may stack on bottom G-tetrad.

We further determined the structure of WT22m G4(II) on the basis of NMR restraints (27) by X-PLOR (Figure 3C and Supplementary Table S2) (31,32,36). The structure of WT22m G4(II) showed a typical (3+1) hybrid G4 conformation with three different types of loop (Figure 3C). Among them, the lateral loop comprised C_4_ and C_7_ residues in CCACC linker exhibited not only an external stacking to the bottom G-quartet (G_3_-G_9_-G_18_-G_22_) but also a hydrogen bonding within C_4_ and C_7_ residues (Figure 3D). These results provide a rational explanation of why C_4_ or C_7_ mutation of WT22m affecting the stability of WT22m G4(II) would favor the G4(I) conformation, implying the importance of a single base mutation that may lock a G4 structure without further conversion.

Very recently, the influence of pH on the potential formation of hydrogen bonds between cytosine bases in G4 structures has been documented (37,38). Thus, we have conducted both WT22m and WT22m-T4 at pH 5 to examine the possible C:C+ base pairs. NMR spectra showed no change of an imino proton signal near 13 ppm in WT22m-T4 while an imino proton signal near 15 ppm was detected in WT22m at pH 5 (Supplementary Figure S7A), not only indicating a formation of C:C+ base pairing by the protonated C_4_ or C_7_ but also confirming the C_4_ and C_7_ bases in close proximity. In addition, CD spectra of WT22m showed larger contrast between 295-nm and 265-nm signals at pH 5 (Supplementary Figure S7B) than at pH 7 (Figure 1), implying less G4(I) formation at pH 5. Moreover, the Tm measured by CD melting curves showed no appreciable difference for WT22m-T4 but about 2.8°C increase for WT22m at pH 5, indicating that C:C+ base pairing could further stabilize G4(II).

### Kinetic studies of structural change from G4(I) to G4(II)

**Figure 4.**
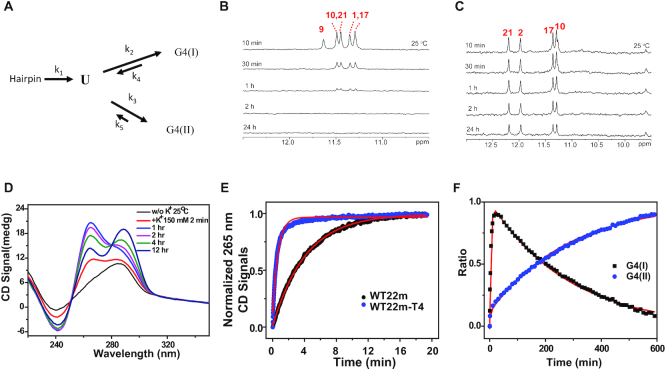
Kinetic and thermodynamic study of conformational change of WT22m. (**A**) Four-state model starting from hairpin (Hp) to G4(I), and G4(II) via an ensemble unfolding state (U) between G4(I) and G4(II). NMR HDX kinetics of (**B**) WT22m-T4 and (**C**) WT22m. The sample was in Tris-buffer, and lyophilized after addition of 150 mM K^+^ overnight for WT22m-T4 and WT22m, and then dissolved in 99% D_2_O immediately before NMR measurement at 10 min, 30 min, 1 h, 2 h and 24 h. (**D**) Time-dependent CD spectra of WT22m after addition of 150 mM K^+^ at 25°C. (**E**) The kinetic trace of WT22m and WT22m-T4 by monitoring the 265-nm CD signal after addition of 150 mM K^+^ at 25°C (**F**) The kinetic trace of WT22m by monitoring the 265-nm CD signal for G4(I) (black dot) and 295-nm signal for G4(II) (blue dot) after addition of 150 mM K^+^ at 25°C were fitted to a four-state kinetic model (red line).

Time-dependent imino proton NMR spectra of WT22m revealed three distinct secondary structures, Hp, G4(I) and G4(II) after the addition of 150 mM K^+^. The absence of the imino proton signals near 13 ppm after the addition of K^+^ suggested that the reversed process to the Hp state can be neglected in the transition model. We further assumed that the transition between G4(I) and G4(II) states involves unfolding intermediate states. Here we tentatively use the state U to represent an ensemble of all the intermediate states between these two transition states. Accordingly, a simplified four-state transition model is proposed to describe the transition kinetics from the initial Hp state to the final G4(II) state via the intermediate G4(I) state of WT22m after the addition of 150 mM K^+^ (Figure 4A). The analytical solution for this transition model by using A for Hp, B for G4(I), C for G4(II) and D for U can be found elsewhere (Supplementary Data).

NMR hydrogen-deuterium exchange (HDX) spectra provide a powerful tool to investigate the unfolding kinetics of G4. This is because the HDX rate of each imino proton signal reveals the dynamics of individual hydrogen bond within the G-quartets upon solvent exposure and provides the unfolding kinetics. It is believed the longer the exchange time, the more protected the hydrogen bond. NMR HDX results of WT22m-T4 showed longer exchange time around 25 min on the G_1_, G_10_, G_17_ and G_21_ signals in the middle G-quartet with respect to other guanine signals in the external G-quartets (Figure 4B). However, NMR HDX results of WT22m showed much longer exchange time around 3800 min on the G_2_, G_10_, G_17_ and G_21_ signals in the middle G-quartet of G4(II) (Figure 4C). We also used imino proton NMR spectra to monitor the decay of the G4(I) and the growth of the G4(II) after the addition of K^+^. Typical plots of the decay of G_16_ of G4(I) and the arising time of G_21_ of G4(II) showed similar time constants around 250 min (Supplementary Figure S8A). The 250±50 min time constant was also found for other imino proton signals (Supplementary Figure S8B). Notably, the 25 min HDX time of the G4(I) is very different from the 250 min decay time of the G4(I) of WT22m. This is because the HDX time only counts the G4 unfolding without considering the G4 refolding.

Time-dependent CD spectra showed a rapid growth of the 265-nm signal followed by a slow decay of the 265-nm signal together with a slow growth of the 290-nm signal (Figure 4D). Because of the long collecting time (ca. 8 min) for one imino proton NMR spectrum, the initial growth of CD signal at 265 nm was monitored to determine the arising time of WT22m G4(I). The arising time was ca. 4 min, which was obtained by using a single exponential parameter to fit the arising curve up to 20 min (Figure 4E). In addition, the arising time for the growth of the CD signal of WT22m-T4 at 265 nm was <1 min under the same condition (Figure 4E). The difference of the arising time between WT22m G4(I) and WT22m-T4 G4 is likely due to the unfolding of the Hp structure of WT22m. Previously, we found that the unfolding rate of the Hp structure is likely the rate-determining step of the G4 formation of WT22 after the addition of K^+^ (27). Thus, we considered that the 4 min arising time for the initial growth of G4(I) is the unfolding time of the Hp structure of WT22m. Using four-state transition model with experimental data of k_1_ = (4 min)^−1^ = 0.25 min^−1^, k_4_ = (25 min)^−1^ = 0.04 min^−1^, and k_5_ = (3800 min)^−1^ = 2.6 × 10^−4^ min^−1^, we were able to fit the CD signals at 265 nm for the growth and decay of G4(I) and 290 nm for the growth of G4(II) of WT22m after the addition of 150 mM K^+^ as a function of time up to 600 min (Figure 4F). The curve fitting allowed us to extract the transition rates of 0.23 min^−1^ for k_1_, 533 min^−1^ for k_2_, 50 min^−1^ for k_3_, 0.037 min^−1^ for k_4_ and 2.7 × 10^−4^ min^−1^ for k_5_. The higher rate constant of k_2_ for G4(I) than k_3_ for G4(II) suggested that G4(I) is the initial kinetic product, while the much lower rate constant of k_5_ for G4(II) than k_4_ for G4(I) suggested that G4(II) is the major final product.

### Cytosine modification for structural selection between G4(I) and G4(II)

**Figure 5.**
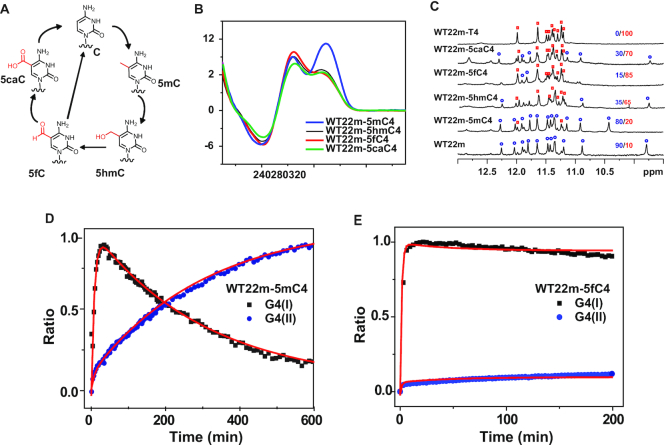
(**A**) Brief scheme of cytosine modification cycle in DNA methylation and demethylation. (**B**) CD spectra of WT22m-5mC4, WT22m-5hmC4, WT22m-5fC4 and WT22m-5caC4 in 150 mM K^+^ solution overnight at 25°C. (**C**) Imino proton NMR spectra of WT22m, WT22m-5mC4, WT22m-5hmC4, WT22m-5fC4, WT22m-5caC4 and WT22m-T4 in 150 mM K^+^ solution overnight at 25°C. The population ratios were estimated by the integration of the peak volumes of imino protons, G16 and G18 (G4(II) (blue)/G4(I) (red)). The kinetic trace of (**D**) WT22m-5mC4 and (**E**) WT22m-5fC4 by monitoring the CD 265-nm signal for G4(I) (black dot) and 295-nm signal for G4(II) (blue dot) after addition of 150 mM K^+^ at 25°C were fitted to a four-state kinetic model (red line).

It is known that cytosine modification, such as 5-methylcytosine (5mC) for DNA methylation together with 5-hydroxylmethylcytosine (5hmC), 5-formylcytonsine (5fC), and 5-caboxylcytosine (5caC) for DNA demethylation, plays critical role in epigenetic regulation (39,40). These naturally occurring bases generated by a reversible cycle of oxidative chemical reaction via specific enzyme are highly associated with the gene expression in cancer, embryo development and other epigenetic diseases (Figure 5A) (40–42). Considering the crucial effect of C_4_ and C_7_ in stabilizing G4(II) structure of WT22m, it is curious to examine whether these cytosine analogues could play an active role in G4 structure because of the significant impact of cytosine modification in epigenetic regulation. Since the substitution of C_4_ by T_4_ in WT22m could lock the WT22m G4(I) state without further conformational transition to the G4(II) state, we investigated the effect of cytosine modification of 5mC, 5hmC, 5fC and 5caC at the C_4_ residue on the G4 structure of WT22m. Interestingly, the CD spectra of WT22m-5hmC4, −5fC4 and −5caC4 showed a major band at 265 nm together with a minor band at 295 nm, while the CD spectra of WT22m-5mC4 showed a major band at 295 nm together with a minor band at 265 nm (Figures 5B). The relative populations of the G4(I) and G4(II) structures for these sequences with each modification at the C_4_ residue can be estimated from NMR spectra (Figure 5C). The population for WT22m-T4 G4 structure is found to be ∼10% in WT22m and ∼20% in WT22m-5mC4, but ∼85% in WT22m-5fC4, ∼65% in WT22m-5hmC4 and ∼70% in WT22m-5caC4, indicating that cytosine demethylation significantly increases the population of WT22m G4(I) structure. In addition, CD melting results showed similar Tm for WT22m-5hmC4, −5fC4 and −5caC4 to the 69.2°C for WT22m-T4 G4, but the same *T*_m_ for WT22m-5mC4 to the 73°C for WT22m G4(II) (Supplementary Figure S9 and Table 1).

We further examined NMR HDX spectra of both G4(I) and G4(II) states in WT22m-5fC4. The HDX time of this G4(I) is ca. 35 min, which is similar to the ca. 25 min HDX time of WT22m G4(I). However, the HDX time of this G4(II) is dramatically reduced to ca. 40 min, which is much shorter than the ca. 3800 min HDX time of WT22m G4(II) (Supplementary Figure S10). In contrast, the HDX time of WT22m-5mC4 G4(II) did not show such large difference from that of WT-22m G4(II) (Supplementary Figure S11). Time-dependent CD spectra were further conducted to monitor the kinetic traces of G4(I) and G4(II) in WT22m-5mC4 and WT22m-5fC4 (Supplementary Figure S12). The CD results of WT22m-5mC4 showed a rapid growth of the 265-nm signal followed by a slow decay together with a slow growth of the 290-nm signal (Figure 5D), which are similar to the CD results of WT22m. However, the CD results of WT22m-5fC4 showed a rapid growth of the 265-nm signal without significant decay at longer time (Figure 5E), implying that the G4(I) structure is dominating. Using the experimental data obtained for k_1_, k_4_ and k_5_ for the curve fitting based on four-state transition model, we could obtain five transition rates for WT22m-5mC4 and WT22m-5fC4 (Table 2). The key finding is that cytosine modification plays a dramatic effect on the unfolding rate of G4(II) in WT22m-5fC4, indicating that the 5fC modification at the C_4_ residue of WT22m could destabilize G4(II) conformation by disrupting loop interaction.

**Table 2.** The kinetic rate constants, k_1_, k_2_, k_3_, k_4_ and k_5_ of topological conversion in WT22m, WT22m-5mC4 and WT22m-5fC4 G4 inducing by K^+^

	k1 (min^−1^)	k2 (min^−1^)	k3 (min^−1^)	k4 (min^−1^)	k5 (min^−1^)
WT22m	0.23 ± 0.03	533 ± 173	50 ± 20	0.037 ± 0.010	2.7 ± 0.9 × 10^−4^
WT22m-5mC4	0.12 ± 0.01	1173 ± 777	147 ± 83	0.029 ± 0.010	1.7 ± 0.9 × 10^−4^
WT22m-5fC4	0.58 ± 0.14	653 ± 120	73 ± 27	0.027 ± 0.010	0.023 ± 0.006

## DISCUSSION

Similar type of this peculiar G4 structure as WT22m G4(I) has been documented in the *chl1* sequence d[GGGTGGGGAAGGGGTGGGT] and in the s23 sequence d[GGGTAGGGCAGGGGACACAGGGT] of human papillomavirus (HPV) in K^+^ solution (43,44). However, the detection of a dominant CD band at 265 nm in WT22m-T4 is different from the detection of two CD bands at 295 and 265 nm in *chl1* (Supplementary Figure S13) and s23 (44). It is noteworthy that the hydrogen-bonding directionality of G-tetrads is all clockwise for WT22m-T4 but involves two clockwise and one counter-clockwise for *chl1* and s23. Previously, Dickerhoff and Weisz demonstrated that a fluoride-modified guanine can favor anti glycoside conformation leading to flipping the hydrogen-bonding directionality of a G-tetrad (45). The reported data supported the idea that the 265- and 295-nm bands in the CD spectra for a G4 structure are mainly determined by hydrogen-bonding directionality, instead of G4 structure (46). The formation of a (3+1) hybrid G4 structure with the same hydrogen-bonding directionality of G-quartets by using modified guanine residues (xanthine and 8-oxoguanine) (47) also led to the observation of a distinct 265-nm signal in its CD spectrum which is generally detected in the parallel G4s (48). Therefore, the detection of the CD band at 265 nm can be described by the same clockwise hydrogen-bonding directionality, although WT22m-T4 does not adopt an all-parallel-stranded G4 conformation. The flipping of the hydrogen-bonding directionality of the bottom G-tetrad in WT22m-T4 as compared to the counterpart in *chl1* might be related to the structural contexts next to the quadruplex-stem loop junction (G_2_ and G_9_) and V-shaped loops (G_16_ and G_20_).

Previous studies showed numerous conformational changes between two different G4 structures, such as from an antiparallel form in Na^+^ solution to hybrid form after the addition of K^+^ (23,49–50), from different nonparallel forms to parallel form (51,52), from a chair-type antiparallel form to two hybrid forms (53), or from a hybrid-II form to hybrid-I form (24). However, it is surprising that the atypical G4 structure could play an intermediate state for the conformational change from an Hp structure to a typical G4 structure, introducing a new possible scenario in G4 folding pathway. Notably, the melting temperature of WT22m-T4 G4 was only 3.8°C lower than that of WT22m G4(II). However, the HDX time of the imino protons of WT22m-T4 G4(I) was much shorter than the counterpart of WT22m G4(II). Similar results were also observed in the comparison between WT22m-5mC4 and WT22m-5fC4. It is possible to have more transient states in the conformational change between G4(I) and G4(II), which is out of our detection limit. Nevertheless, our kinetic results indicate that a parallel-like G4 structure can act as a kinetic trapped state, which is very different from the concept of G4 folding.

Here, inducing C:C+ base paring between C_4_ and C_7_ under acidic condition (pH 5), could not only reduce the formation of G4(I) but also increase thermal stability (Supplementary Figure S7), indicating that C:C+ base pairing could stabilize G4(II). Similar finding has been previously reported in the study of two major G4 structures of a single substitution of dG with 8Br-dG at position 21 of d[(GGGGCC)_3_GGGG] (37). However, the preference of G4(II) structure in case of WT22m might not necessarily be limited to the C_4_-C_7_ stabilizing interaction. For instance, G4(I) has 2-nt (C_12_ and A_13_) edgewise loop across a wide groove, which is likely to be unfavorable. In addition, it is noted that the difference between G4(I) and G4(II) folding topologies is significant, and it is not only involving the rearrangement of the (GTCACC or CCACC) edgewise loop. Hence, we cannot attribute the difference in the two structure stabilities solely based on the comparison between G_3_-C_8_ and C_4_-C_7_ base pairs in their corresponding loops.

It is known that cytosine modification by either methylation or demethylation could play an important role in regulating gene expression (39,40). For example, 5mC is generally associated with the inhibition of gene expression, whereas 5hmC is normally associated with the increase of gene expression (40–42). However, there have been only a few studies on the effect of these cytosine analogs on DNA structure. Previously, Balasubramanian group showed that 5fC can convert the B-form double helix DNA to the F-form and the reduction of 5fC to 5hmC can reverse F-form back to B-form (54). Such structural conversion induced by cytosine modification provides an example for a potential structural effect of DNA methylation and demethylation and further results in different epigenetic consequences.

Considering the hydrogen bonding between the amino groups of C_4_ and C_7_ involved in WT22m, it is possible that the oxygen in hydroxyl group of 5hmC and carbonyl group of 5fC and 5caC in modified WT22m are capable of forming a relatively stable intramolecular hydrogen bond and impedes the formation of intermolecular hydrogen bonding in the lateral loop of G4(II). Indeed, the population of G4(II) structure is much lower in WT22m-5fC4 than in WT22m-5mC4. This is probably because cytosine demethylation at the C_4_ residue plays a dramatic effect on the unfolding rate of G4(II) structure with ca. 100-fold larger in WT22m-5fC4 than in WT22m-5mC4, but minor effect on other transition parameters based on the simulation of four-state kinetic model (Table 2). Thus, a possible explanation is that the intermolecular hydrogen bonding acceptor for carbonyl group of 5fC with the ability as hydrogen bonding donor cause structural destabilization in G4(II). Taken together, these results not only demonstrate that the loop configuration is an additional driving force to maintain certain G4 structure but also highlight that slight chemical modification on nucleotide base could affect G4 structure via remodeling the hydrogen bonding interaction in loop configuration.

In summary, we found an unprecedented G4 structure as an intermediate state in the structural transition from an Hp structure to a final G4 structure of WT22m after the addition of K^+^. Spectral analysis indicated that the structure of this intermediate state can be locked by a single-base mutation and verified as an aberrant parallel G4 structure. It is unusual that an aberrant parallel G4 structure is an intermediate state during the conformational transition to a hybrid G4 structure. In addition, the underlying structural selection of these two G4 structures not only provides the first example of the effect of demethylated cytosine modification on G4 structure but also highlights G4 conformational variation, which may be involved in epigenetic regulation via different cytosine modification.

## DATA AVAILABILITY

PDB: accession codes 6L8M and 6L92.

## Supplementary Material

gkz1207_Supplemental_File
