# Supplementary material for: Cytosine epigenetic modification modulates the formation of an unprecedented G4 structure in the WNT1 promoter
Source: Nucleic Acids Res. 2020 Jan 8;48(3):1120–30. doi: 10.1093/nar/gkz1207 (PMC7026657; doi:10.1093/nar/gkz1207)

**Supporting information**

**Cytosine epigenetic modification modulates the formation of an unprecedented G4 structure in the *WNT1* promoter**

Zi-Fu Wang,^1^ Ming-Hao Li,^1^ I-Te Chu,^1,2^ Fernaldo Richtia Winnerdy,^3^ Anh Tuân Phan,^3,4^ Ta-Chau Chang,^1,2,*^

^1^ Institute of Atomic and Molecular Sciences, Academia Sinica, Taipei 106, Taiwan, R.O.C.

^2^ Department of Chemistry, National Taiwan University, Taipei 106, Taiwan, R.O.C.

^3^ School of Physical and Mathematical Sciences, Nanyang Technological University, Singapore

^4^ NTU Institute of Structural Biology, Nanyang Technological University, Singapore

**Kinetic equation**

**Analytical solutions for the four-state transition model**

The analytical solutions for this transition model (A for Hp, B for G4(I), C for G4(II), and D for U shown in Figure 5A) can be derived as follows:


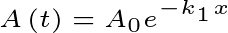


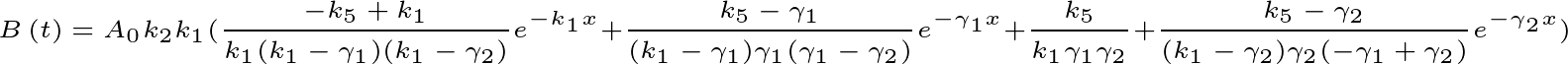


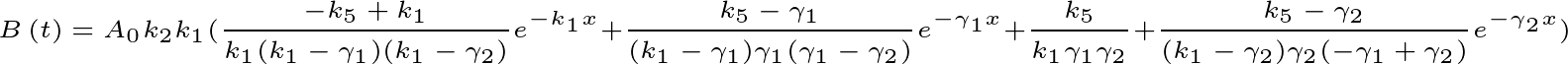


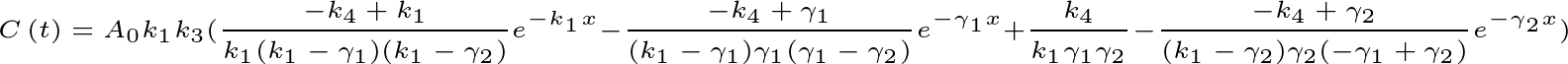


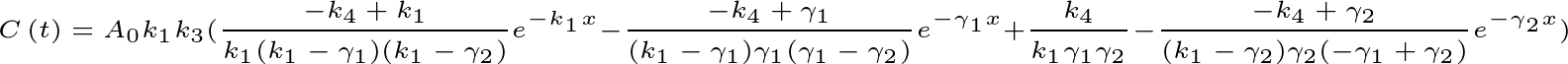


and


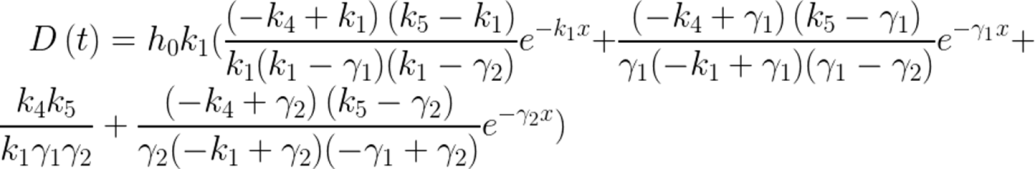


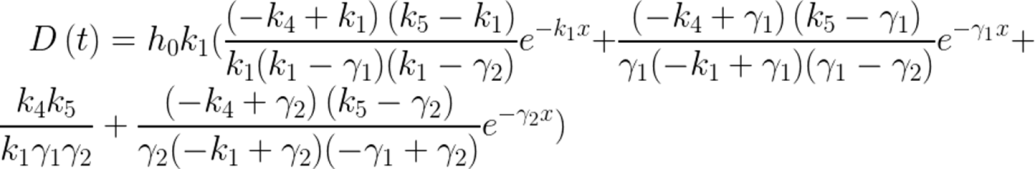


Where γ_1_γ_2_ = k_3_k_4_+k_2_k_5_+k_4_k_5_ and γ_1_ + γ_2_ = k_2_ + k_3_ + k_4_ + k_5_. The initial conditions were A(0) = A_0_, B(0) = C(0) = D(0) = 0. The experimental quantities associated with the G4(I) and G4(II) were extracted from the time-dependent CD spectra, which were used as input for nonlinear regression to extract k_1_, k_2_, k_3_, k_4_, and k_5_ in the Origin 7.5 software (OriginLab Corp., Northampton, MA, USA)

Figure S1. The 1D ^15^N-^1^H SOFAST-HMQC spectrum of 6% ^15^N enriched WT22m-T4 G4 samples at G_3_ together with the imino proton NMR spectrum of WT22m-T4 G4 in the top row.

Figure S2. The 1D ^15^N-^1^H SOFAST-HMQC spectra of 6% ^15^N enriched WT22m-T4 G4 samples for H8 proton region are shown with the assignments and site-specific labeled that corresponds to labeling sites, and the 1H NMR spectrum of WT22m-T4 G4 at H8 proton region was shown in the top row of stacked spectra.

Figure S3. JR-HMBC NMR spectrum of WT22m-T4 G4. Through-bond correlations between guanine imino and H8 protons via ^13^C_5_ at natural abundance.

Figure S4. H1’- H8 proton regions of NOESY spectrum of WT22m-T4 G4 labeled with residue number of aromatic protons. The NOESY spectrum was recorded at 25 ^o^C with a mixing time of 250 ms.

Figure S5. (A) Br-modified 8-deoxyguanine of WT22m-T4 sequences for verification of syn glycosidic conformation. The imino proton NMR spectra of Br-dG_14_, Br-dG_1_, and Br-dG_2_ in the presence of 150 mM K^+^ solution incubated overnight at 25 ^o^C. (B) CD melting curves of WT22m-T4, Br-dG_14_, Br-dG_1_, and Br-dG_2_ in 150 mM K^+^ solution overnight monitored at CD 265 nm signal.

Figure S6. The TOCSY and NOESY spectrum of WT22m. (A) TOCSY spectra of WT22m. The strong correlation of Cytosine H6-H5 labeled with residue number and the weak correlation of C4H4’’-H6 cross peak was marked with asterisks. The intensity of left panel spectrum was increased 16-fold than right panel. (B) H1’- H8/H6 proton regions of NOESY spectrum of WT22m G4 labeled with residue number of aromatic protons. The missing NOEs were marked with asterisks. (C) An Expanded view of NOESY spectrum of C_4_ with other residues. (D) An Expanded view of NOESY spectrum of C_4_ and C_7_ with the bottom G-tetrad. The data were recorded at 25 ^o^C with a mixing time of 50 ms for TOCSY and 250 ms for NOESY.

Figure S7. I-motif base pairing of C_4_ and C_7_ in WT22m G4(II) under pH 5 condition. (A) imino proton NMR of WT22m-T4 (top) and WT22m (bottom) at 5 ^o^C. (B) CD spectra of WT22m-T4 (black line) and WT22m (red line). (C) CD melting curves of WT22m-T4 (black line) and WT22m (red line). CD spectra were conducted in 150 mM K^+^ solution at 25 ^o^C.

Figure S8. (A) The kinetic trace from the peak intensity of the imino proton NMR signals. The decay signal of G_16_ in the G4(I) (square) and the arising signal of G_21_ in the G4(II) (circle) were normalized and fitted by single exponential curve (red). (B) The decay and arising times of imino proton signals in WT22m after the addition of 150 mM K^+^, where each time was obtained by using single exponential parameter to fit the curve of the corresponding imino proton signal intensity after the addition of K^+^.

Figure S9. CD melting curves of WT22m-5mC4, WT22m-5hmC4, WT22m-5fC4, and WT22m-5caC4 in 150 mM K^+^ solution overnight monitored at the CD 290 nm signal.

Figure S10. NMR HDX kinetics of WT22m-5fC4 G4(I) and G4(II). The sample was in Tris-buffer, and lyophilized 30 min for G4(I), and after addition of 150 mM K^+^ overnight for G4(II) and then dissolved in 99% D_2_O immediately before NMR measurement at 10 min, 30 min, 1 h, 2h, and 24 h.

Figure S11. NMR HDX kinetics of WT22m-5mC4 G4(I) and G4(II). The sample was in Tris-buffer, and lyophilized 30 min for G4(I), and after addition of 150 mM K^+^ overnight for G4(II) and then dissolved in 99% D_2_O immediately before NMR measurement at 10 min, 30 min, 1 h, 2h, and 24 h.

Figure S12. Time-dependent CD spectra of WT22m-5mC4 (A) and -5fC4 (B) after the addition of 150 mM K^+^ at 25 ^o^C.

Figure S13. CD spectra of *chl1* sequence d[GGGTGGGGAAGGGGTGGGT] G4 (black line), plotted with WT22m-T4 G4 (red line) in 150 mM K^+^ solution overnight at 25 ^o^C.

Figure S1.


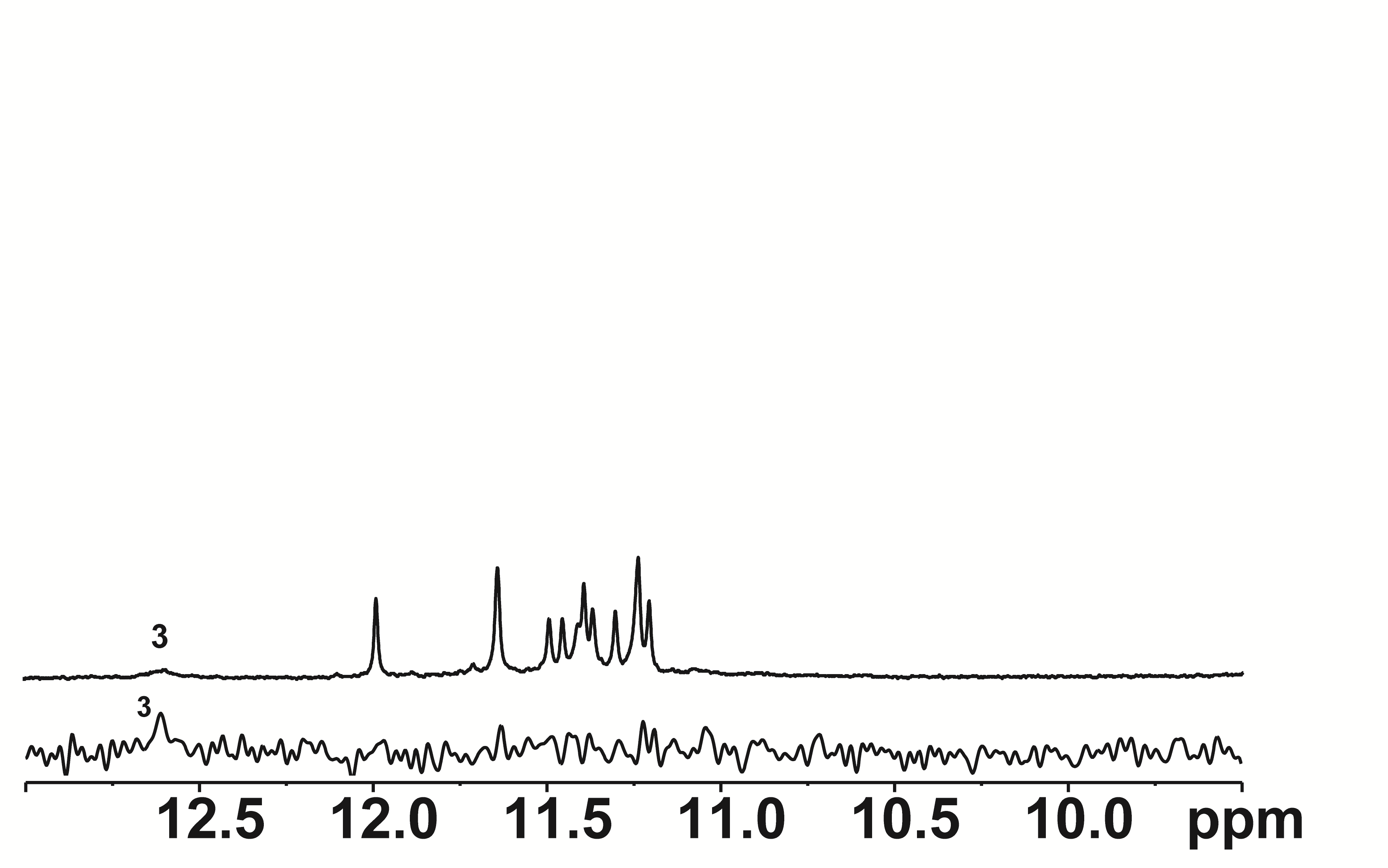


Figure S2.


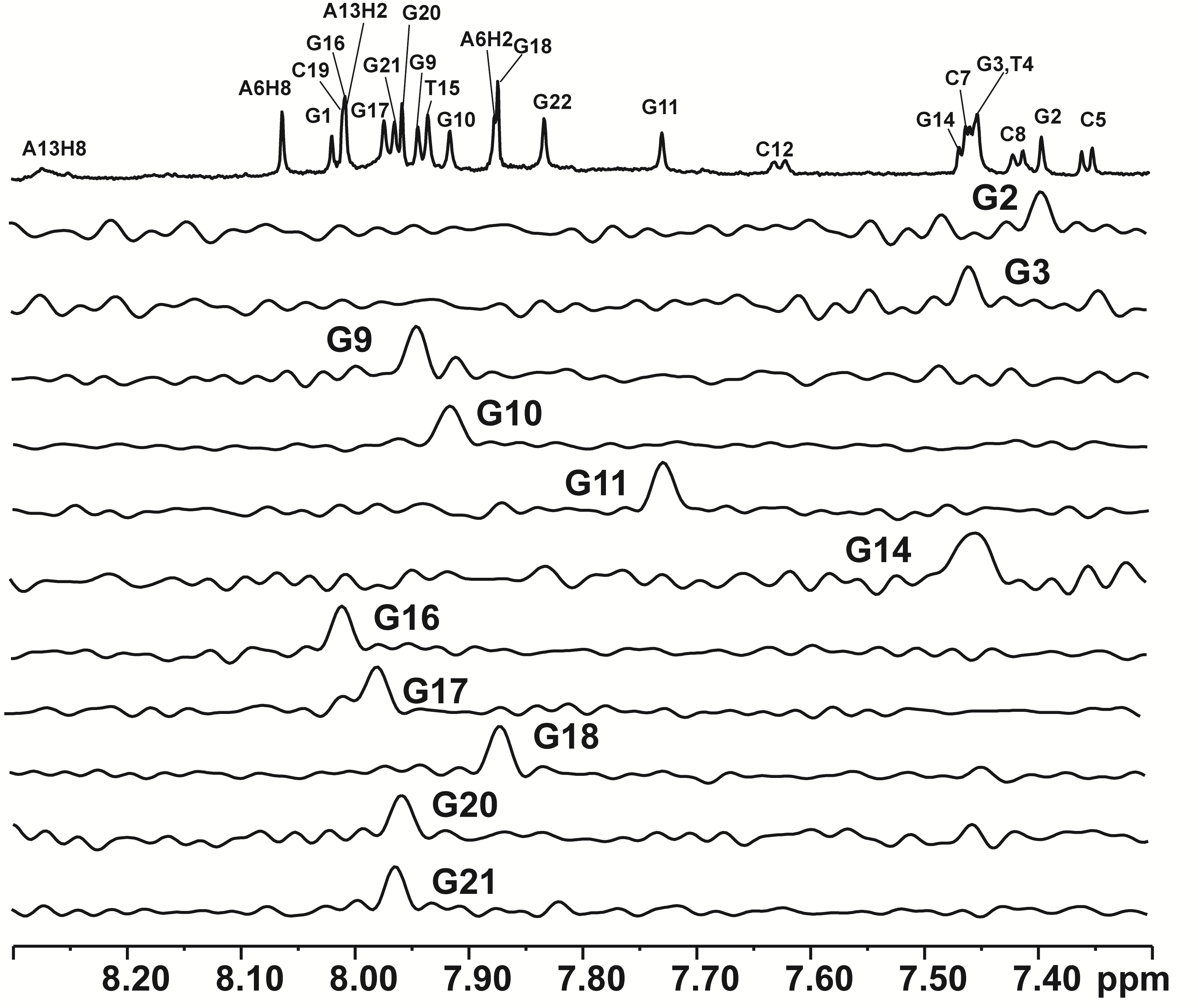


Figure S3.


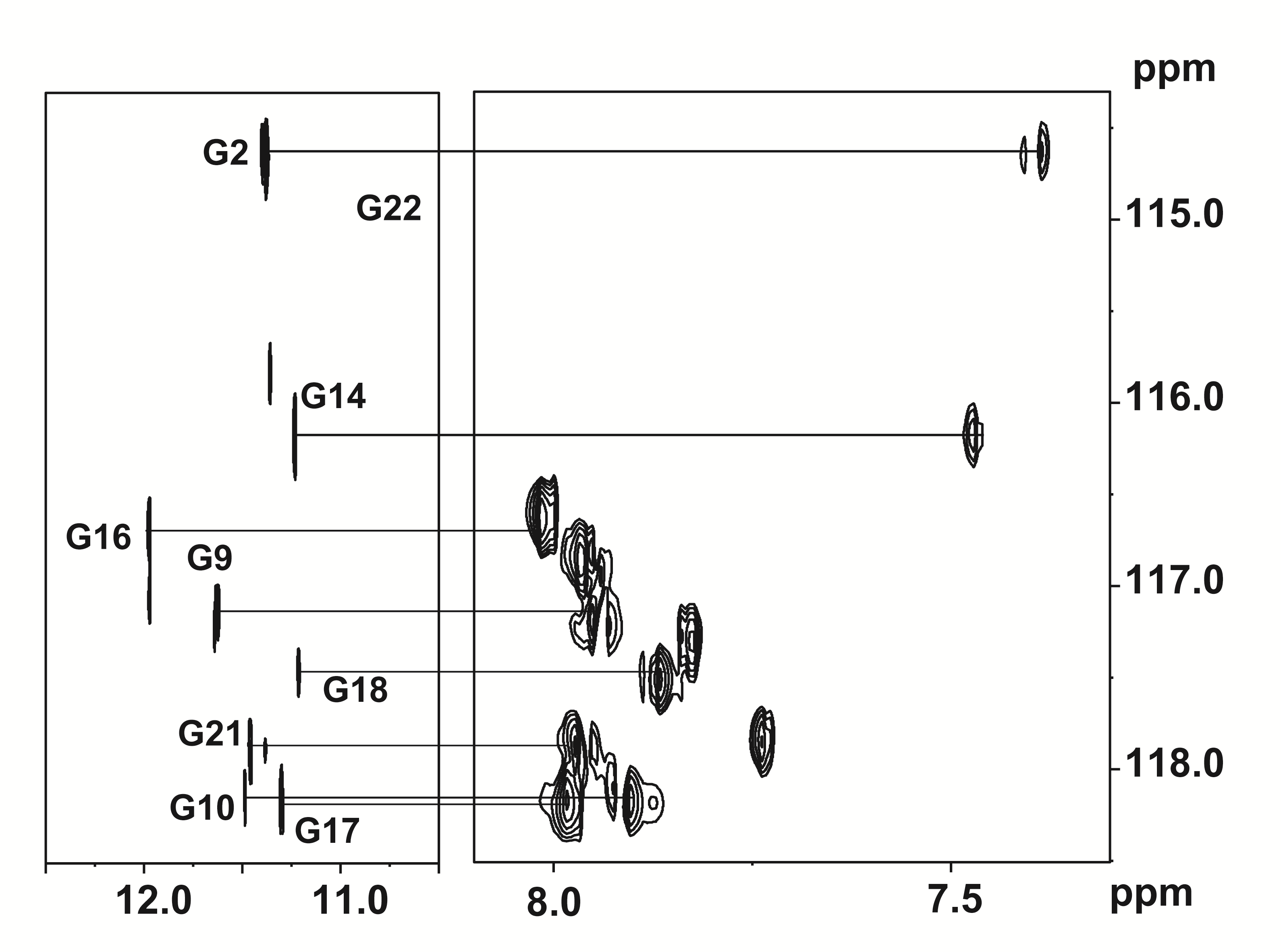


Figure S4.


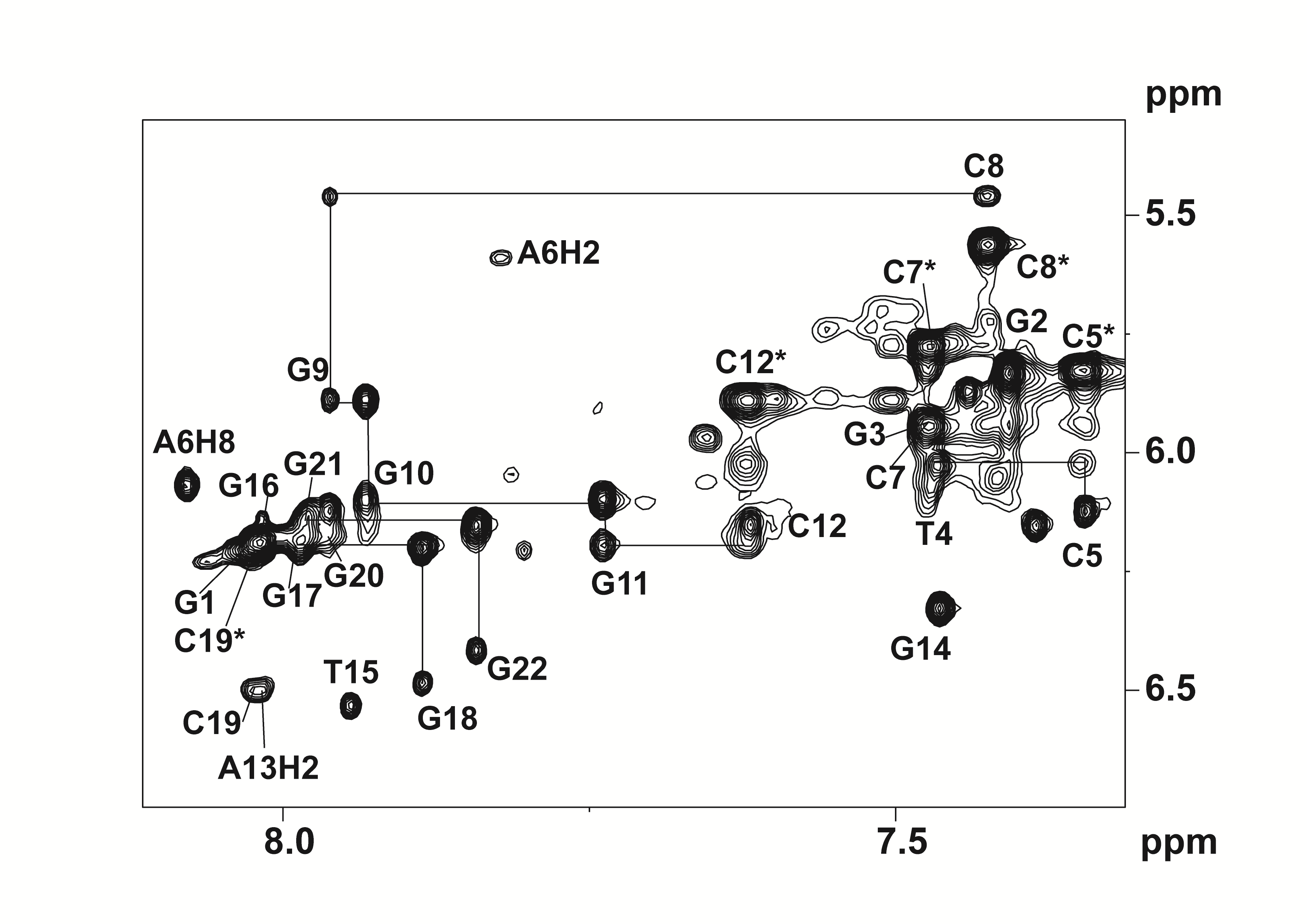


Figure S5.

(A)


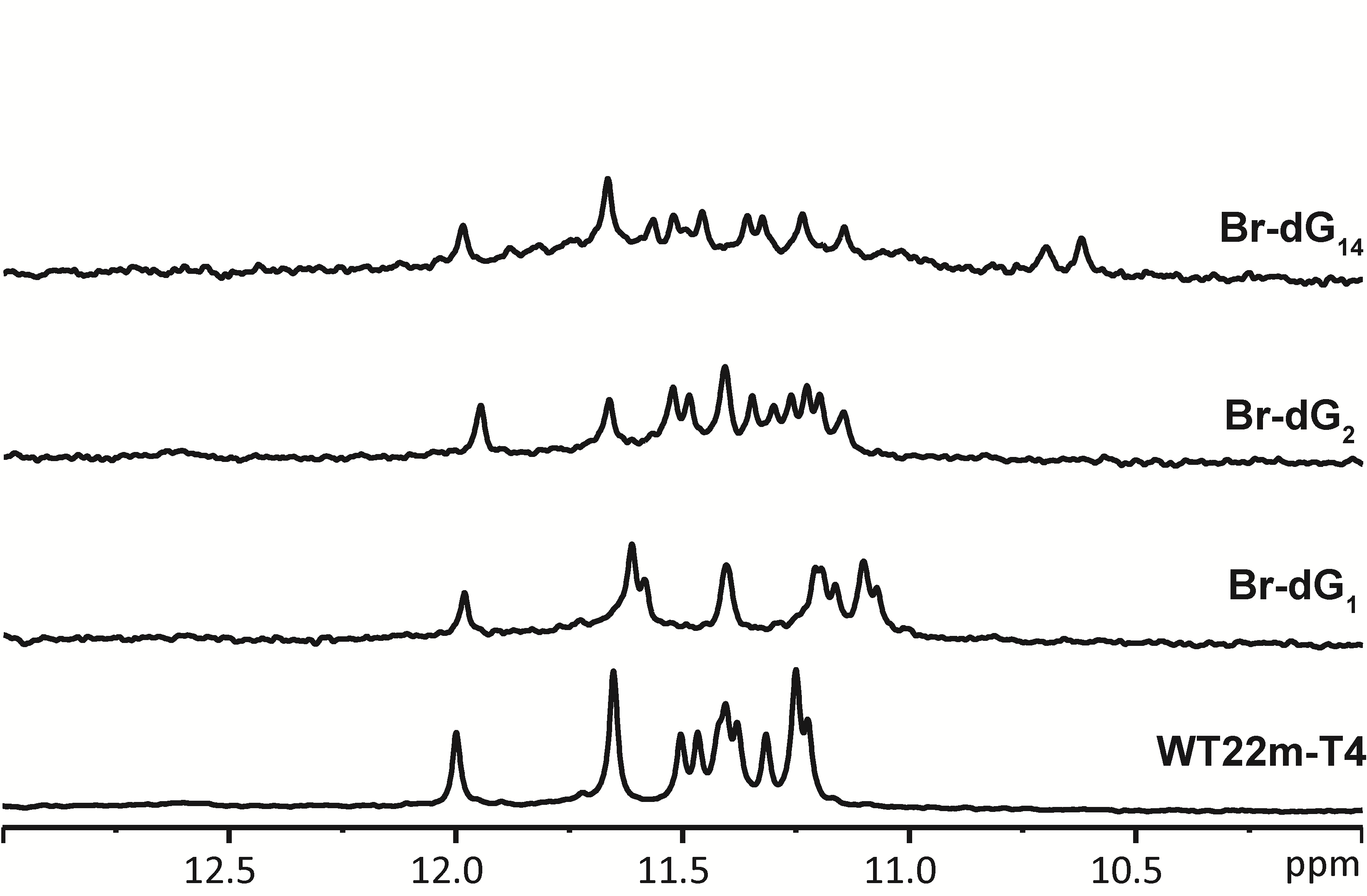


(B)


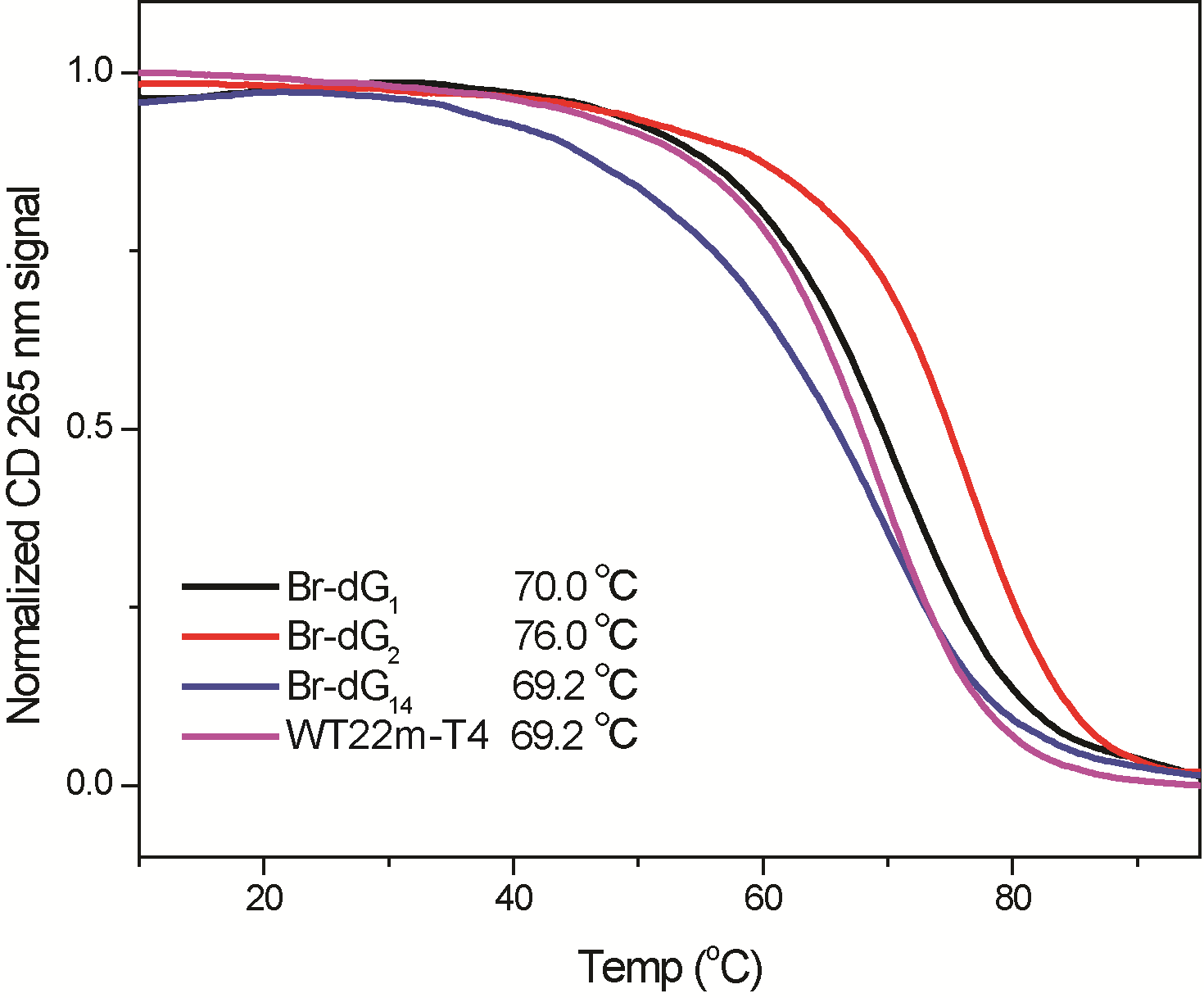


Figure S6.


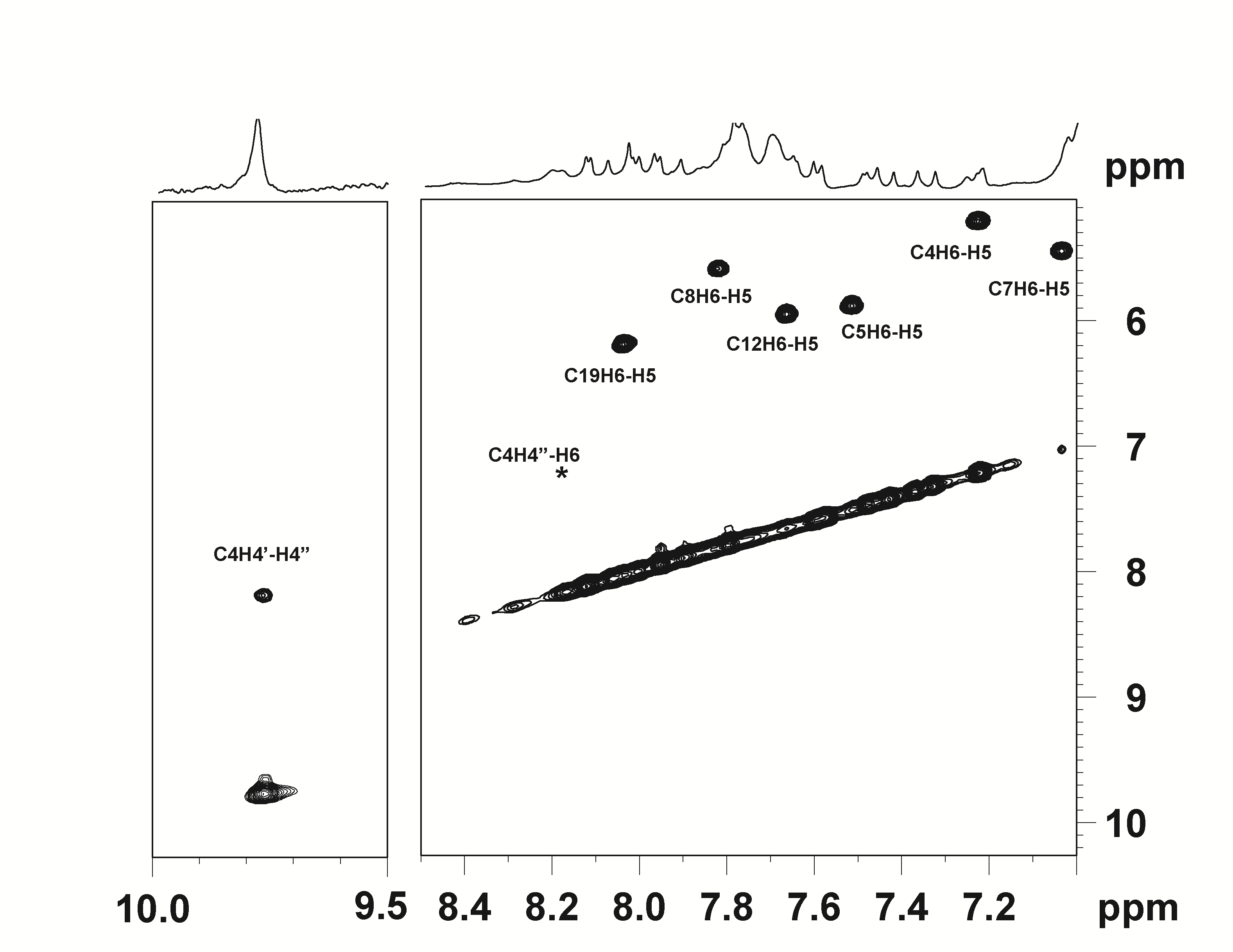
(A)

(B)


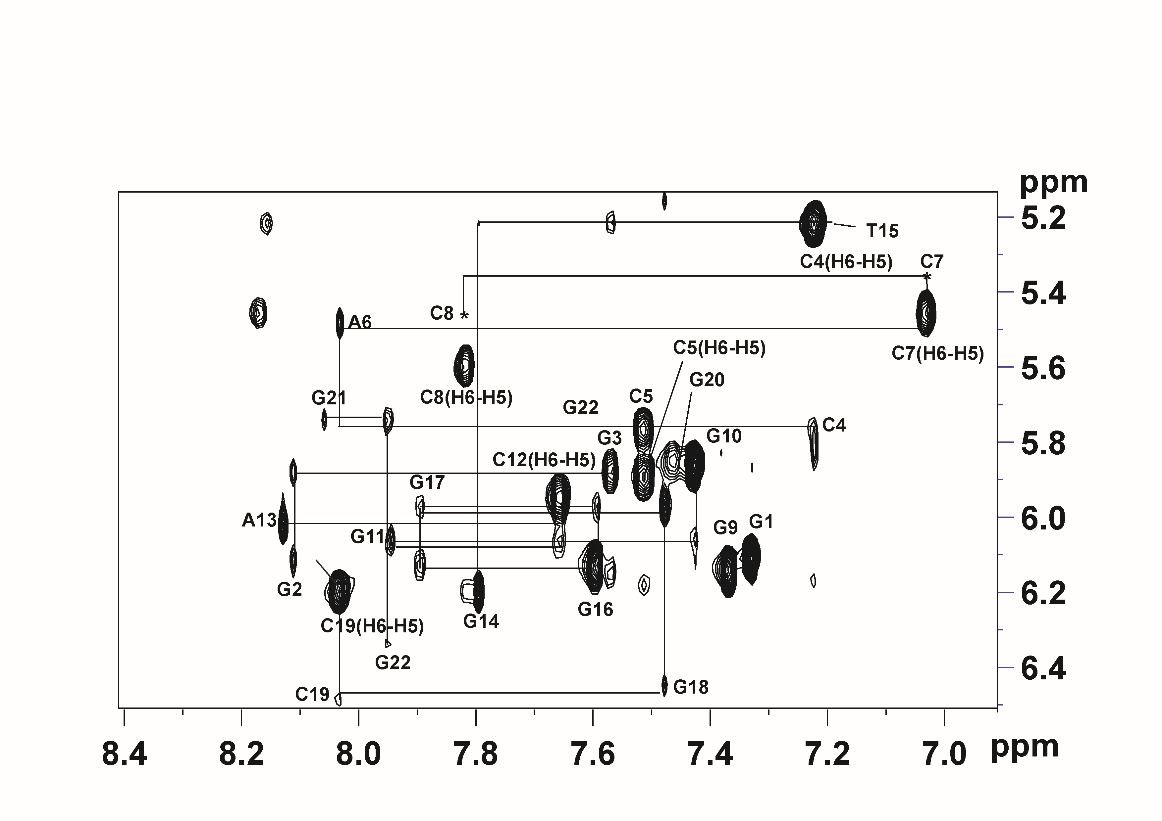


(C)


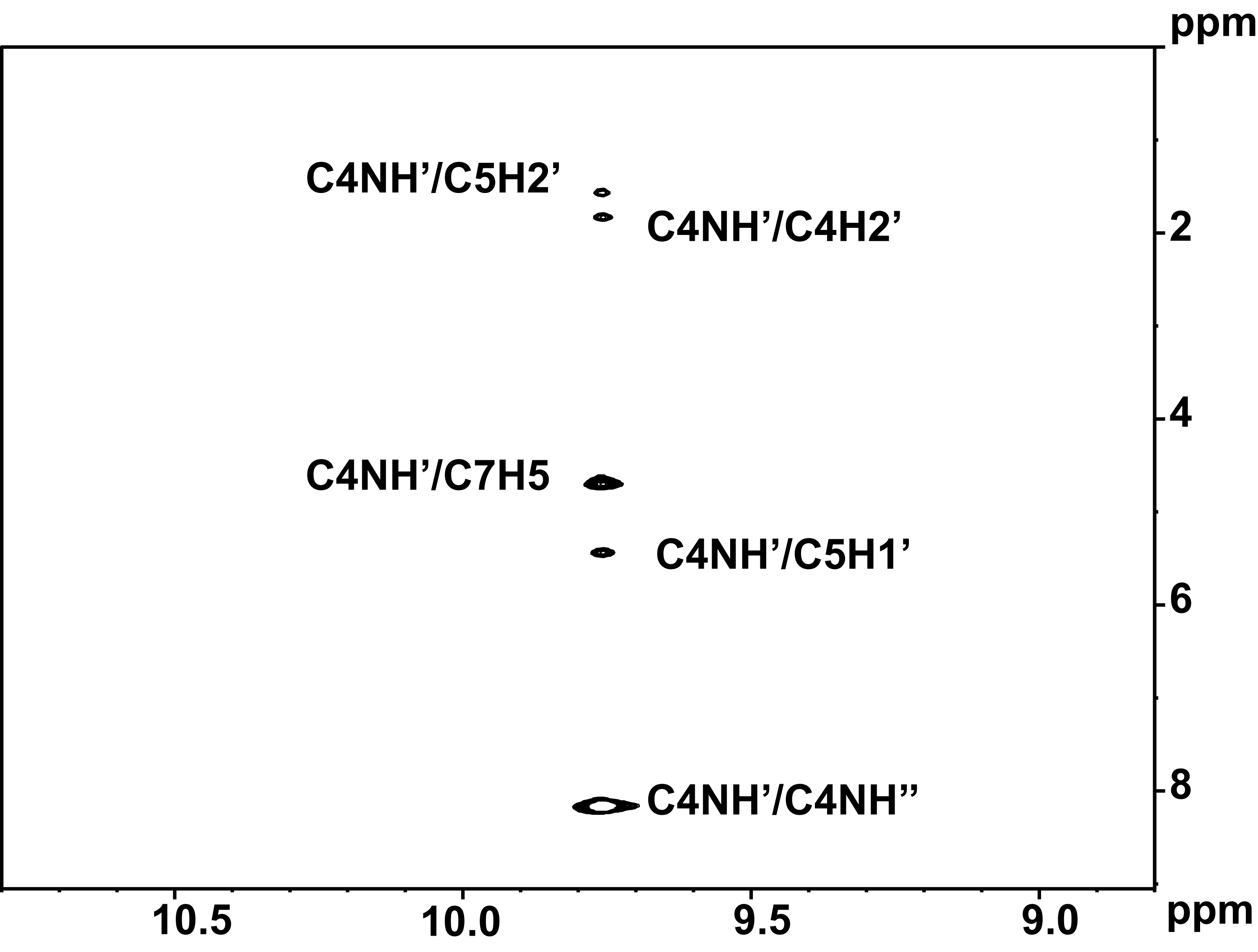


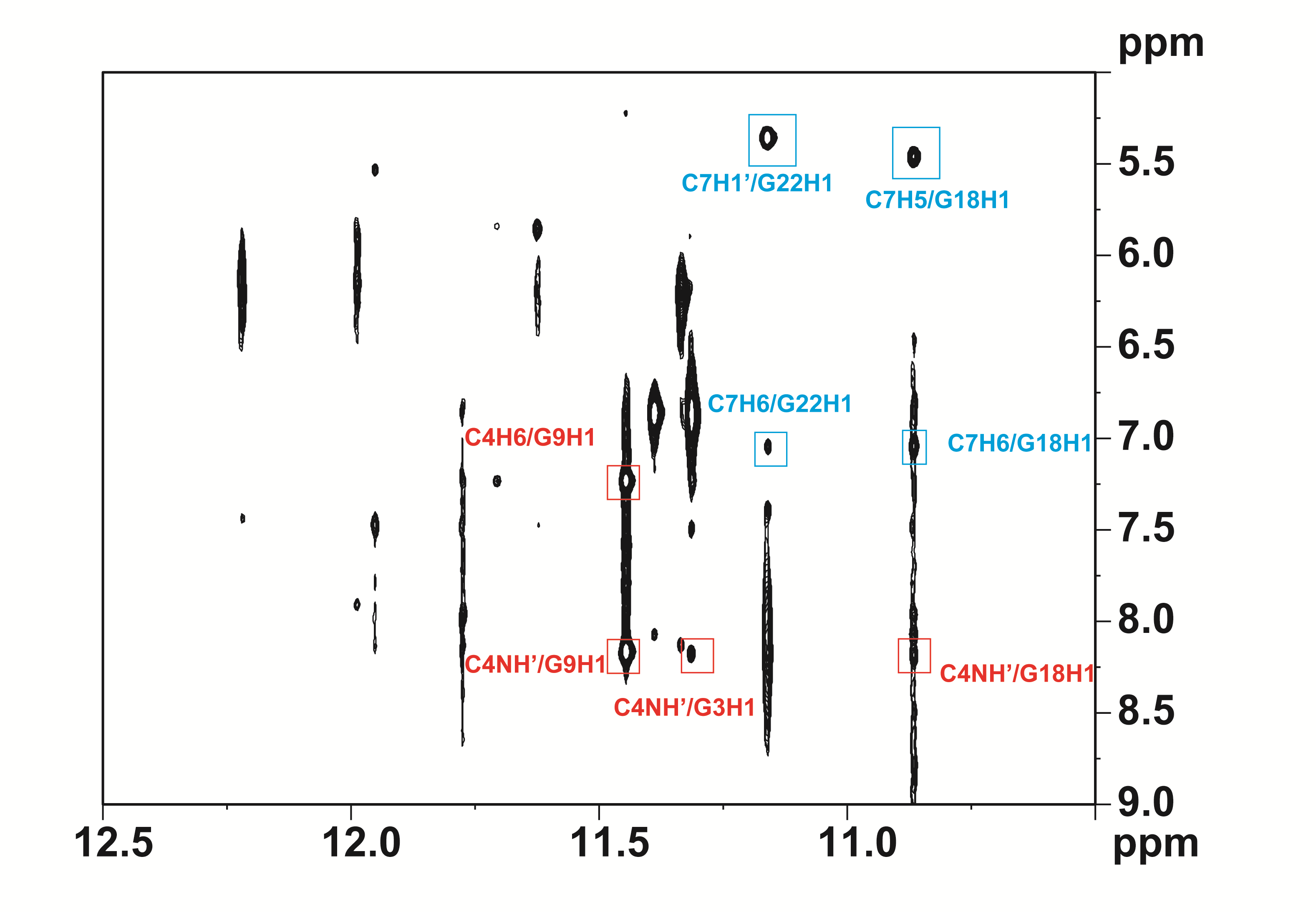
(D)


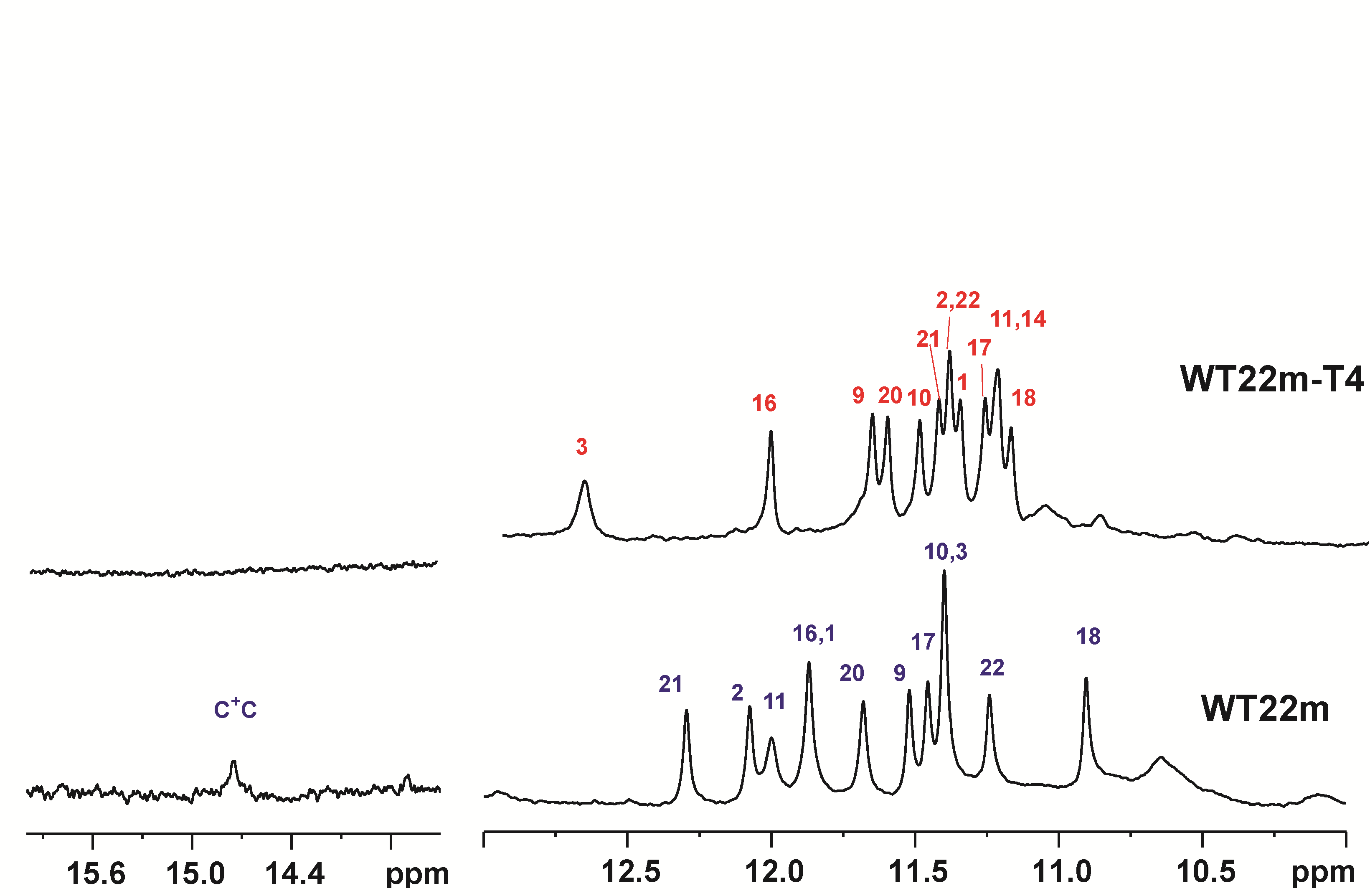
Figure S7.

(A)


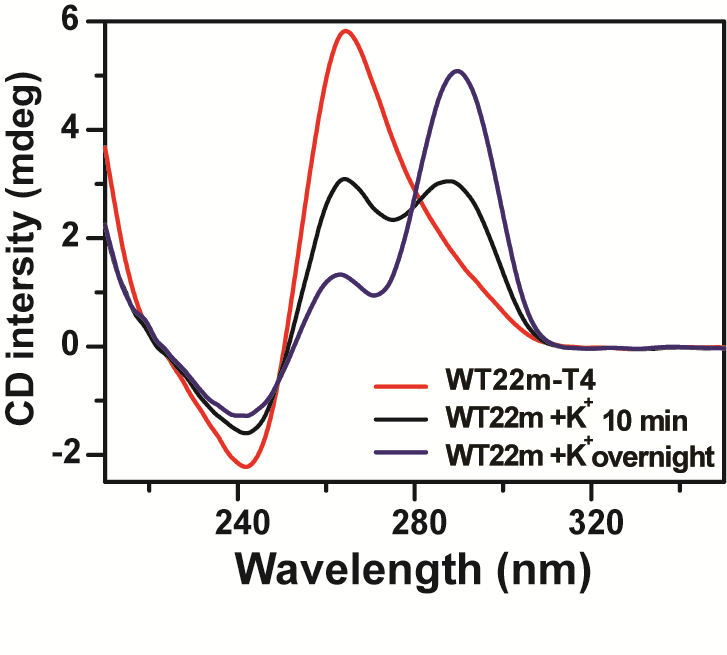
(B) (C)


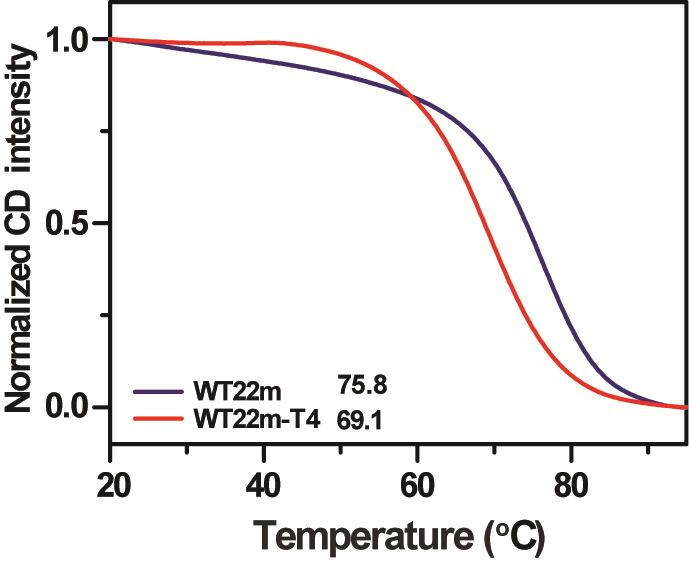


Figure S8.

(A)


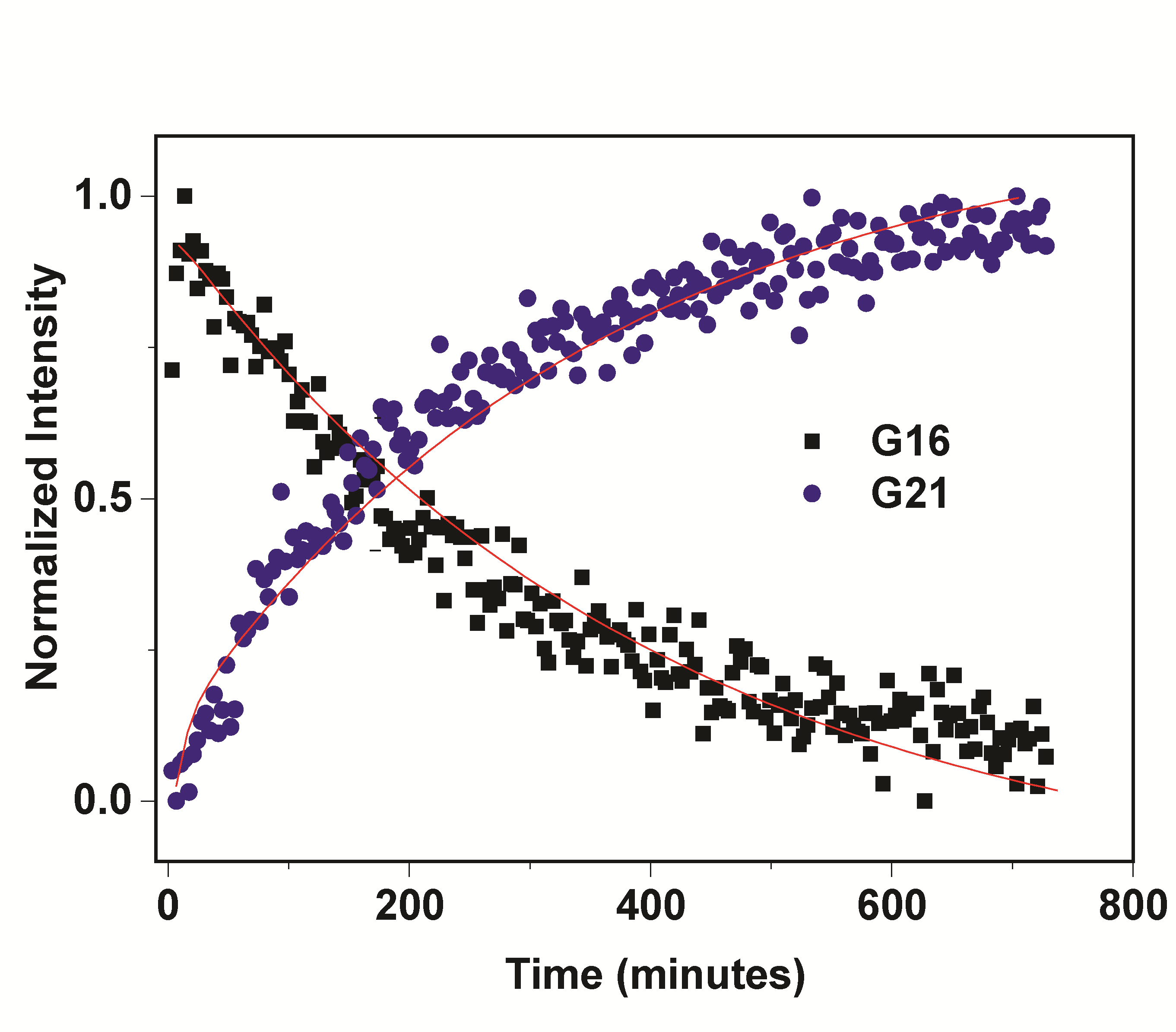


(B)


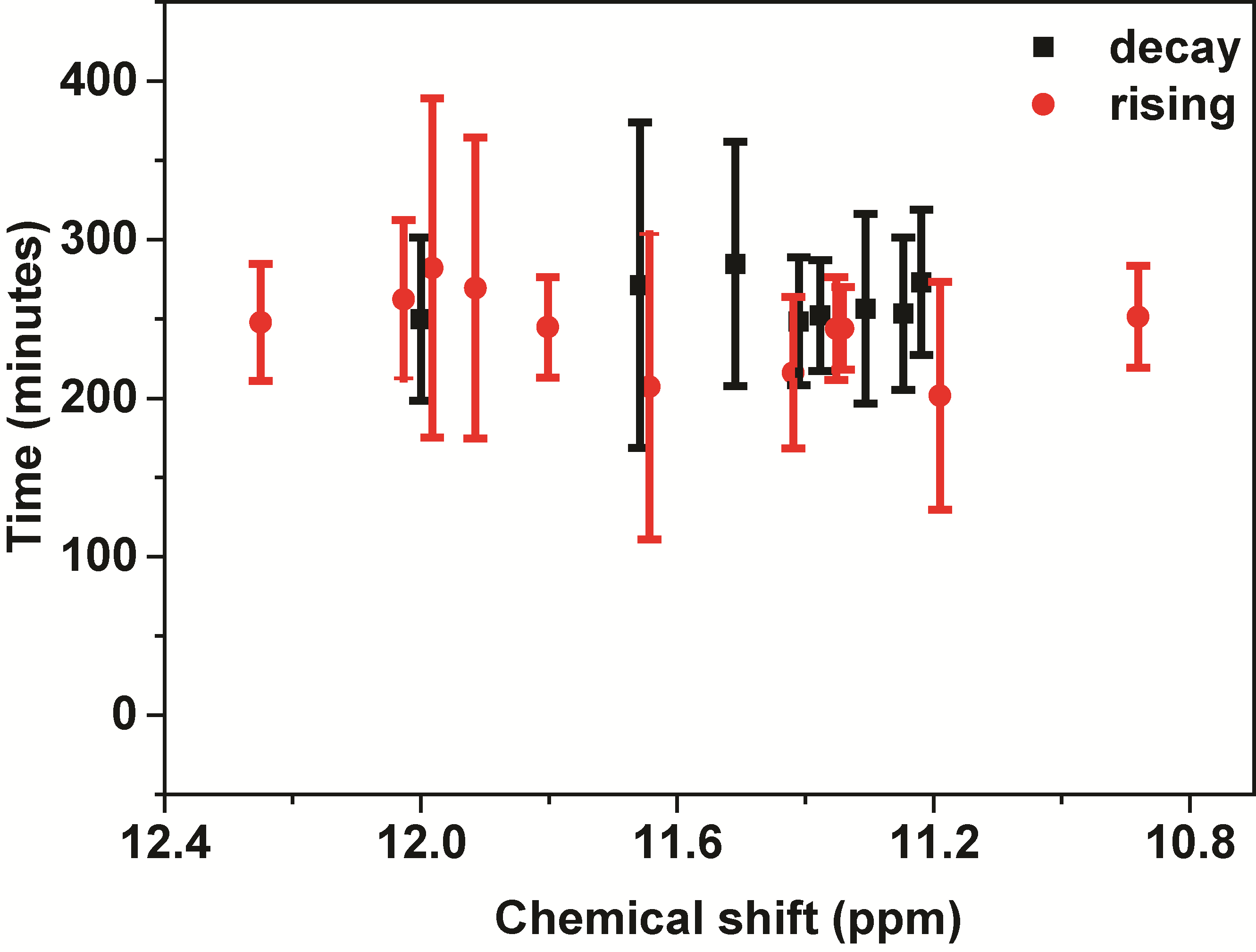


Figure S9.


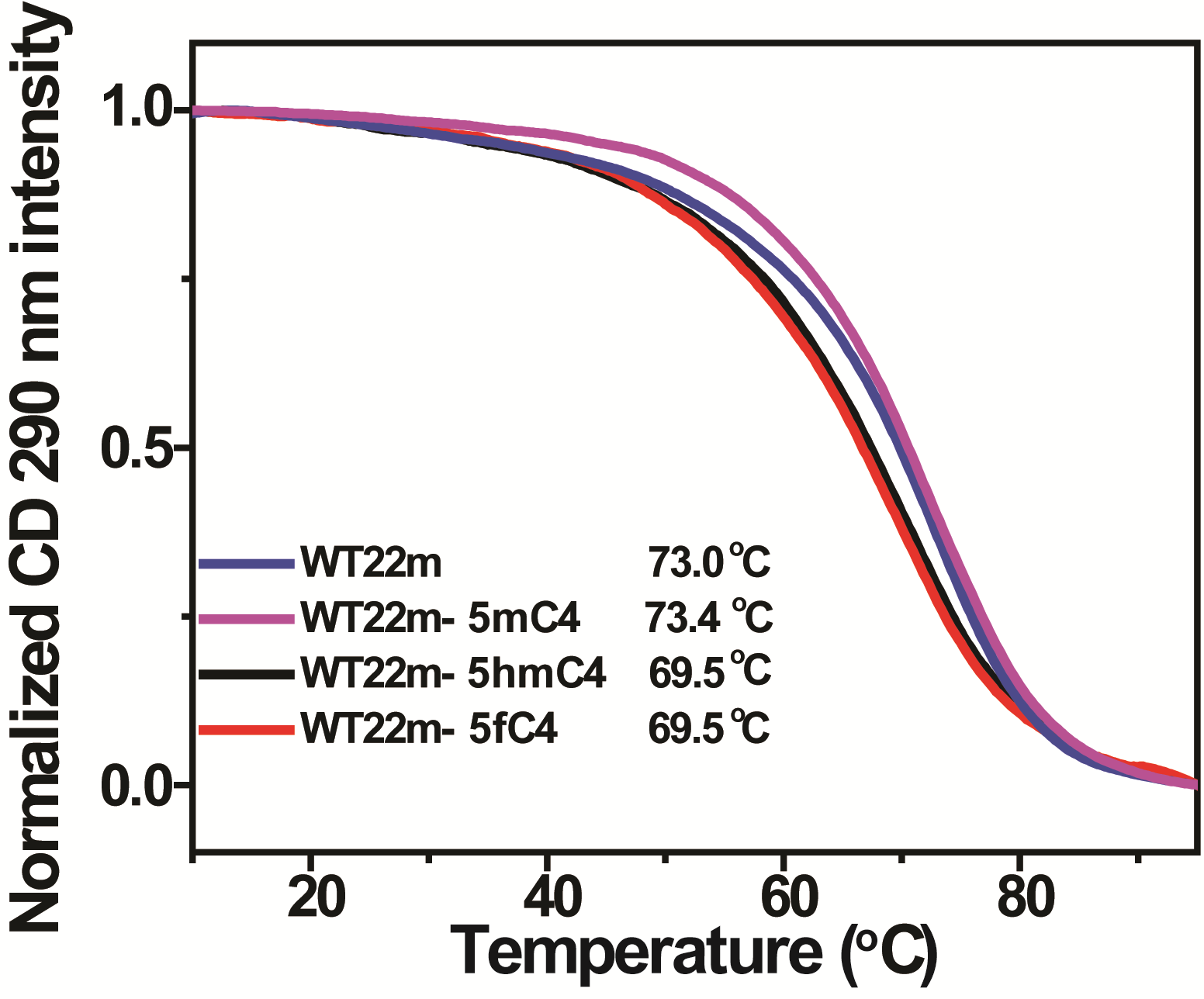


Figure S10.

(A)

WT22m-5fC4


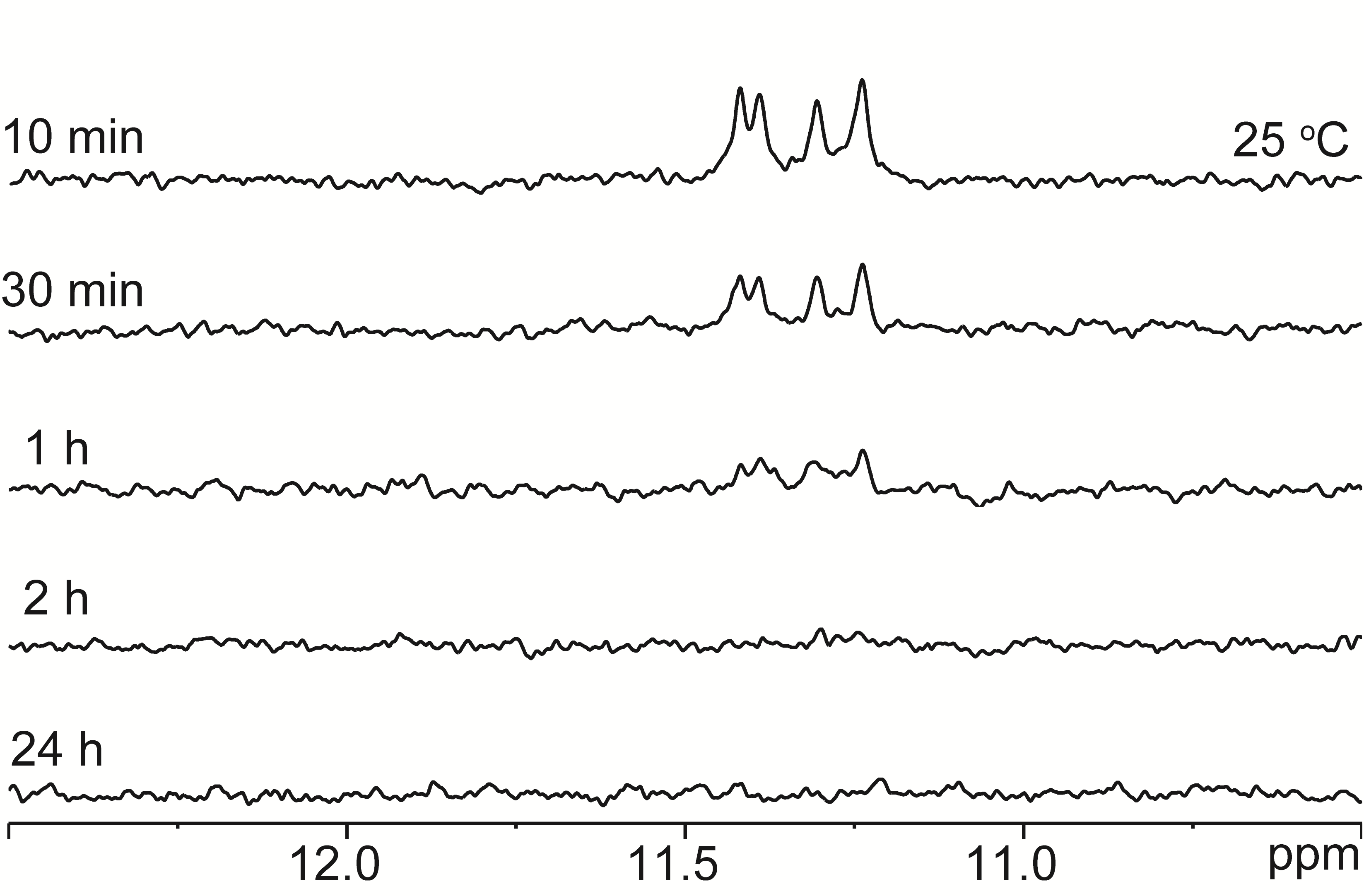


G4(l)

(B)


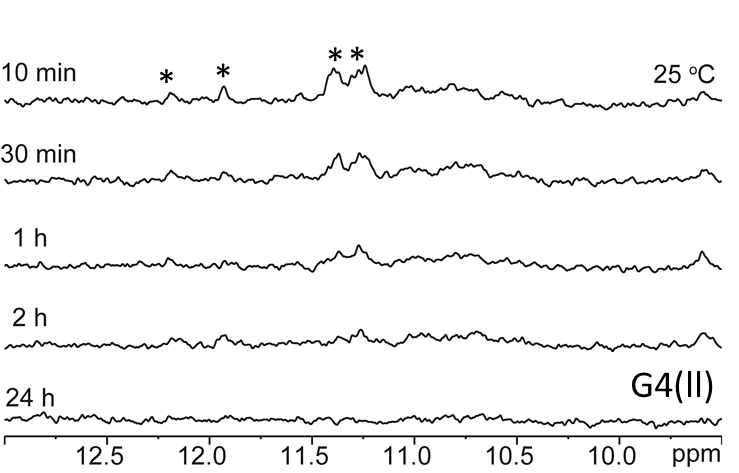


Figure S11.

WT22m-5mC4

(A)


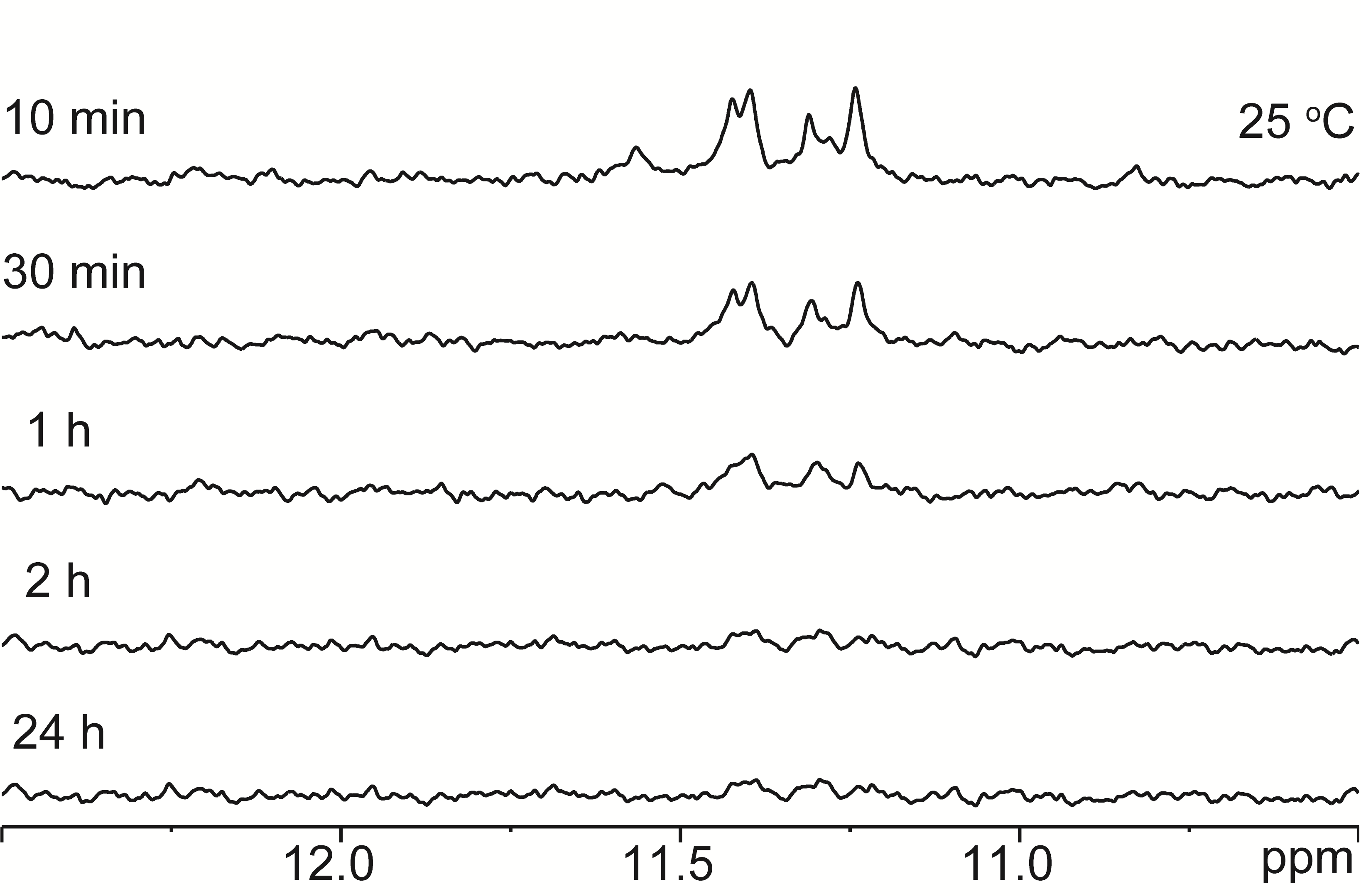


G4(l)

(B)


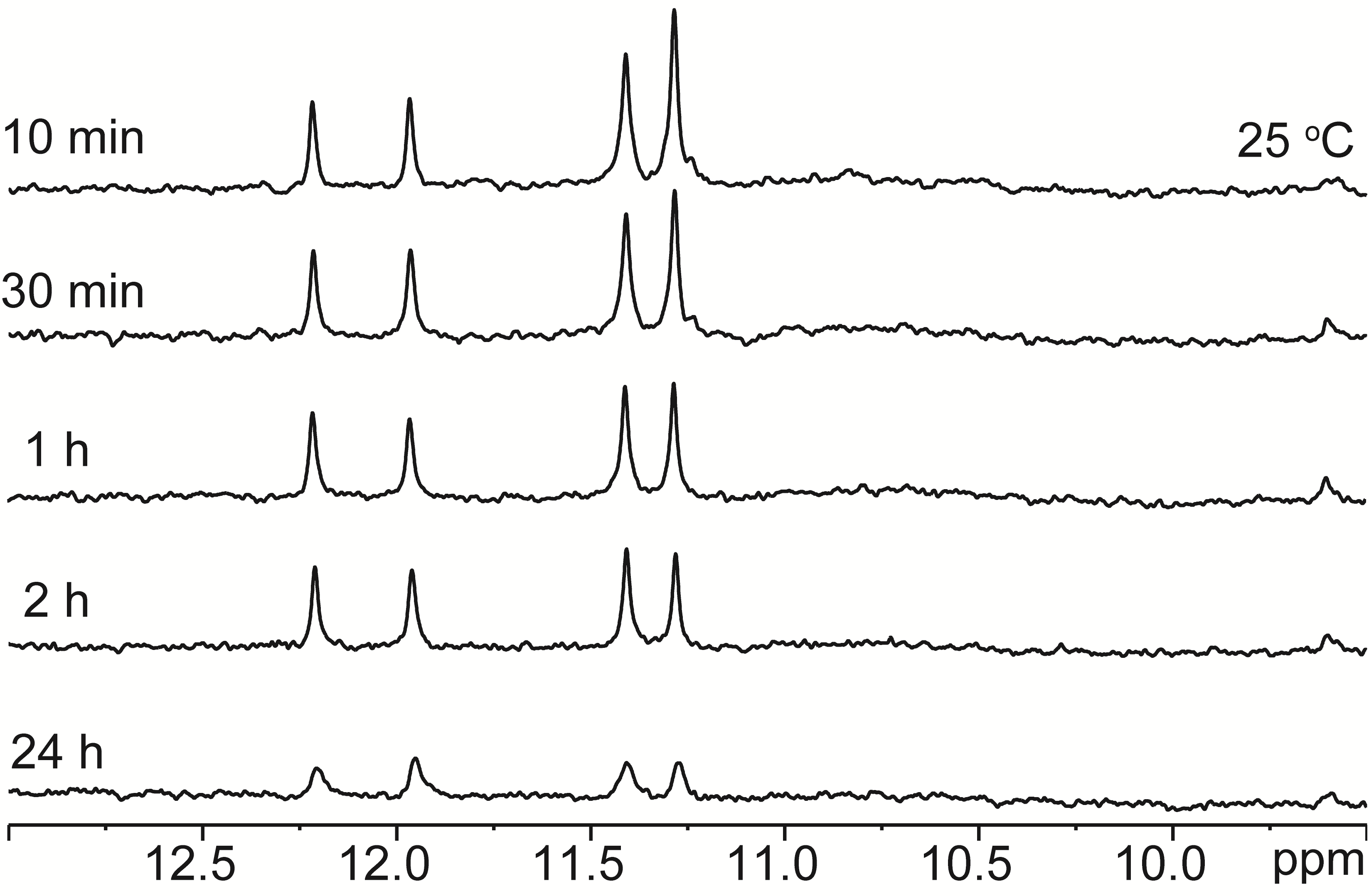


G4(ll)

Figure S12.

(A)


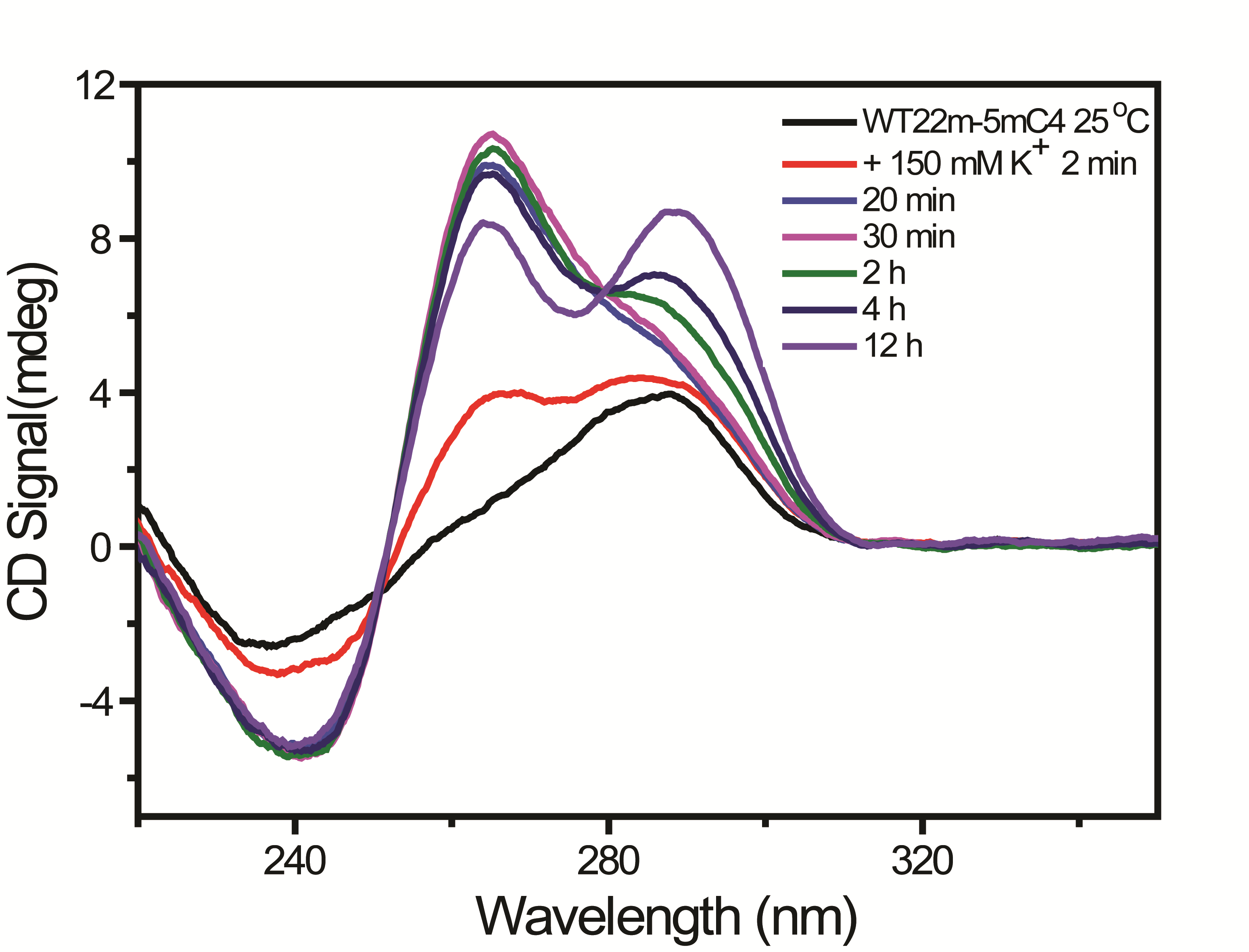


(B)


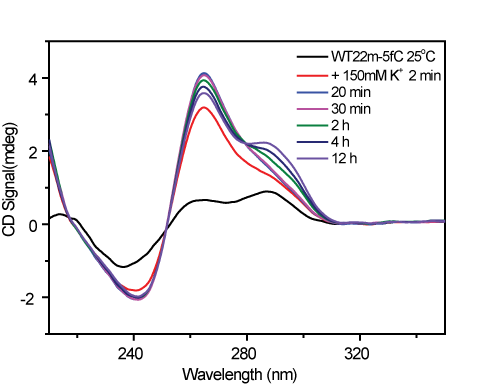


Figure S13.


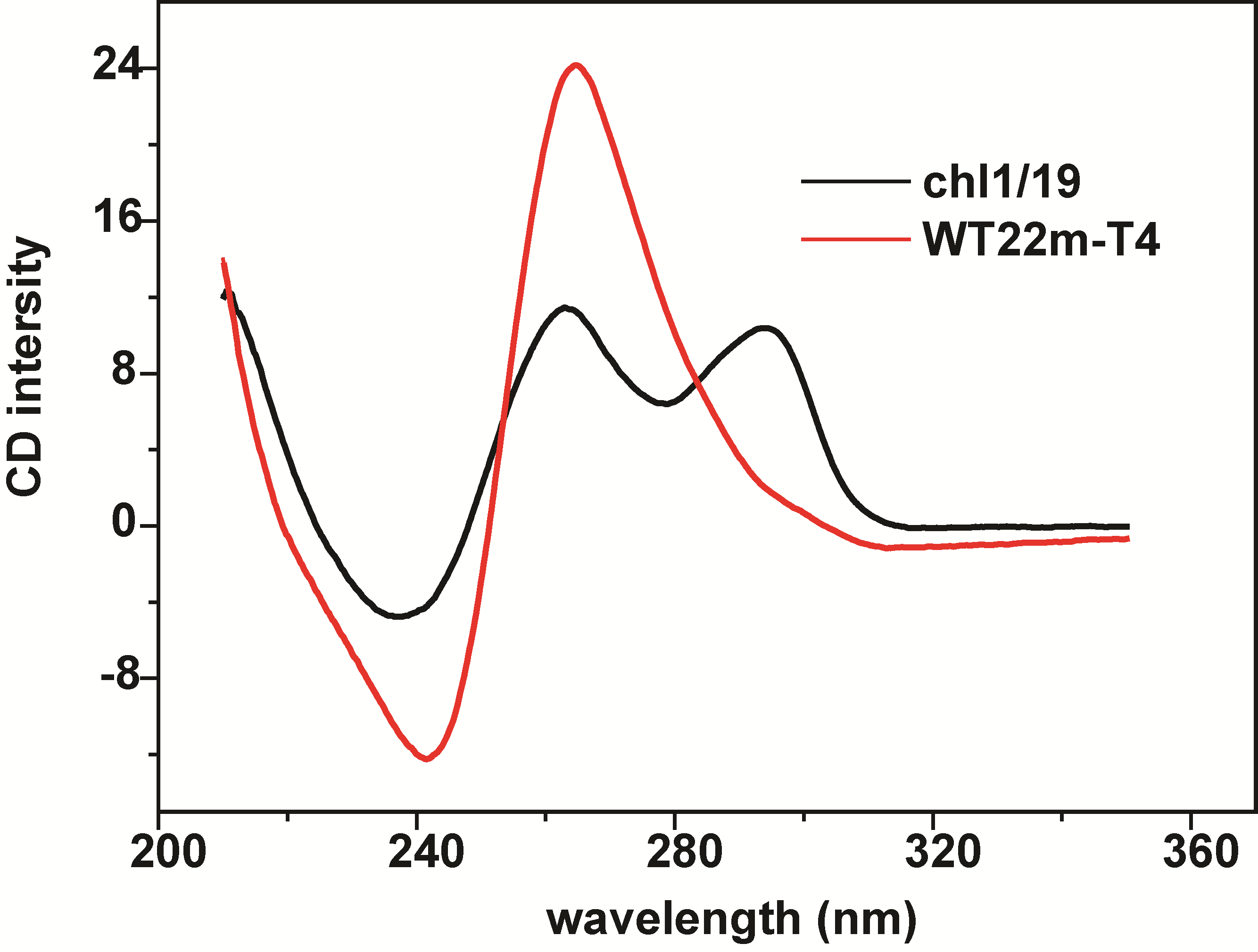


Table S1.


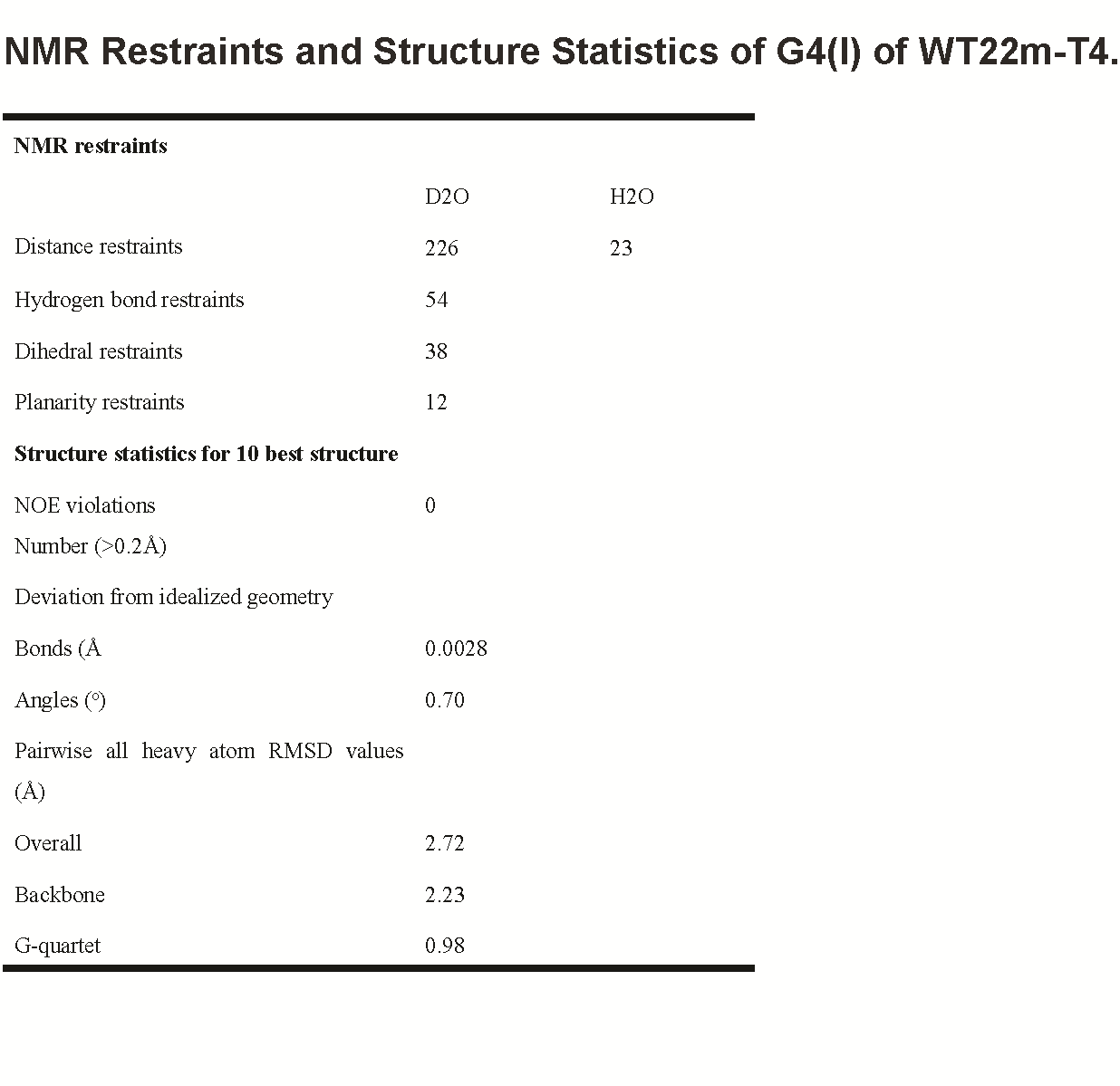


Table S2.


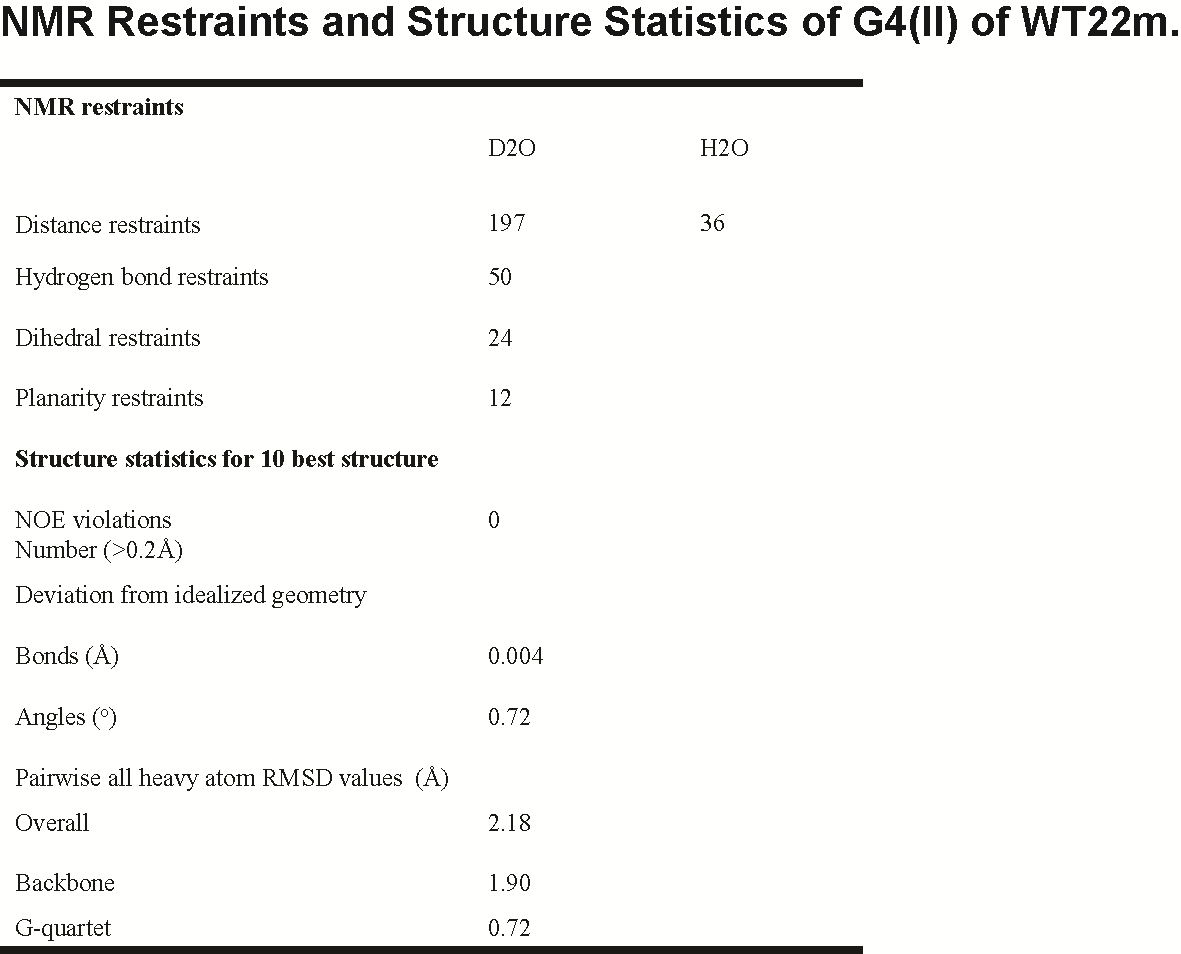

Supplement: gkz1207_Supplemental_File [file gkz1207_supplemental_file.doc]
